# Synergistic Interaction of Rnf8 and p53 in the Protection against Genomic Instability and Tumorigenesis

**DOI:** 10.1371/journal.pgen.1003259

**Published:** 2013-01-31

**Authors:** Marie-Jo Halaby, Anne Hakem, Li Li, Samah El Ghamrasni, Shriram Venkatesan, Prakash M. Hande, Otto Sanchez, Razqallah Hakem

**Affiliations:** 1 Department of Medical Biophysics, University of Toronto and Ontario Cancer Institute, University Health Network, Toronto, Ontario, Canada; 2 Department of Physiology, Yong Loo Lin School of Medicine and Tembusu College, National University of Singapore, Singapore, Singapore; 3 University of Ontario Institute of Technology, Oshawa, Ontario, Canada; Cincinnati Children's Hospital Medical Center, United States of America

## Abstract

Rnf8 is an E3 ubiquitin ligase that plays a key role in the DNA damage response as well as in the maintenance of telomeres and chromatin remodeling. *Rnf8^−/−^* mice exhibit developmental defects and increased susceptibility to tumorigenesis. We observed that levels of p53, a central regulator of the cellular response to DNA damage, increased in *Rnf8^−/−^* mice in a tissue- and cell type–specific manner. To investigate the role of the p53-pathway inactivation on the phenotype observed in *Rnf8^−/−^* mice, we have generated *Rnf8^−/−^p53^−/−^* mice. Double-knockout mice showed similar growth retardation defects and impaired class switch recombination compared to *Rnf8^−/−^* mice. In contrast, loss of p53 fully rescued the increased apoptosis and reduced number of thymocytes and splenocytes in *Rnf8^−/−^* mice. Similarly, the senescence phenotype of *Rnf8^−/−^* mouse embryonic fibroblasts was rescued in p53 null background. *Rnf8^−/−^p53^−/−^* cells displayed defective cell cycle checkpoints and DNA double-strand break repair. In addition, *Rnf8^−/−^p53^−/−^* mice had increased levels of genomic instability and a remarkably elevated tumor incidence compared to either *Rnf8^−/−^* or *p53^−/−^* mice. Altogether, the data in this study highlight the importance of p53-pathway activation upon loss of Rnf8, suggesting that Rnf8 and p53 functionally interact to protect against genomic instability and tumorigenesis.

## Author Summary

DNA double-strand breaks are the most dangerous type of DNA lesions that can occur in cells. Failure to repair these breaks quickly and efficiently can result in either cell death or genomic instability and eventually malignant transformation. Rnf8 and p53 are key proteins in the DNA damage response. We have generated mice that are deficient in both Rnf8 and p53 proteins to determine how these two proteins interact. We found that, when p53 is absent, some of the developmental defects caused by Rnf8 deficiency are rescued. However, this is at the expense of increased chromosomal abnormalities caused by the inability of double-mutant cells to restrain DNA synthesis and cell proliferation and by their escape of cell death in the presence of damaged DNA. We showed that loss of both Rnf8 and p53 led to the accelerated development of both hematological and solid tumors. This study has important implications for human cancer, where mutations in the *p53* gene are found in more than 50% of all cancers but where the other factors that facilitate cancer development are not well understood. This study suggests cooperation between Rnf8 and p53 in the prevention of cancer.

## Introduction

DNA double strand breaks (DSBs) are among the most deleterious type of DNA lesions. They can be generated either by extrinsic factors such as ionizing radiation (IR) or during normal physiological processes such as class switch recombination (CSR) and meiosis [1], [2]. DSBs are highly toxic for cells and failure in the signaling or repair of these breaks can also increase the risk for genomic instability and promote development of diseases including cancer [1], [2]. In response to DSBs, eukaryotic cells have evolved a DNA damage response (DDR) process that involves a network of signaling, repair and checkpoint proteins that lead to either the repair of the damaged DNA or elimination of the damaged cells by inducing their death or entry into senescence [1]. Phosphorylation of H2ax on Ser139 (γ-H2ax) elicited by the sensors of DSBs, Atm and DNA-PK, is an essential event in the initiation of the DDR signaling cascade [1], [2]. This phosphorylation of H2ax prompts the recruitment of Mdc1 to the site of DNA damage where it then undergoes phosphorylation by Atm. Phospho-Mdc1 is then recognized by the E3 ubiquitin ligase Rnf8 *via* its FHA domain [3]. At the DSB sites, Rnf8 cooperates with the E2 ligase Ubc13 to attach K63-linked ubiquitin moieties to chromatin components H2a, H2b and γ-H2ax [4], [5], [6]. This in turn leads to the recruitment of another E3 ligase, Rnf168 which polyubiquitylates H2a-type histones on sites flanking the DSB [7], [8]. Polyubiquitylation of histones leads to the recruitment of DNA damage signaling and repair proteins including 53bp1 and Brca1 [3], [4], [5], [7], [8], [9], [10]. In addition to K63-linked ubiquitylation, in response to DSBs, Rnf8 mediates K48-linked ubiquitylation of JMJD2A leading to its proteasomal degradation and the facilitation of 53bp1 recruitment to DSB sites [11]. Rnf8 also adds K48-linked ubiquitin chains to the non-homologous end joining (NHEJ) protein Ku80, also leading to its proteasomal degradation and facilitating NHEJ mediated DNA damage repair [12]. In addition to its central role in the response to DSBs, Rnf8 was also found to play a role in telomere protection by ubiquitylating and stabilizing the shelterin component Tpp1 at telomere ends [13].

To investigate Rnf8 physiological role, *Rnf8*-null (*Rnf8^−/−^*) mice have recently been generated. These mice harbor various developmental defects as exemplified by their smaller size and male sterility [14], [15], [16]. Cells derived from *Rnf8^−/−^* mice were observed to have increased radiosensitivity, class switch recombination (CSR) defects, and elevated genomic instability [14], [16]. Interestingly, *Rnf8^−/−^* mice also demonstrated an increased risk for tumor development, suggesting that Rnf8 is a *bona fide* tumor suppressor [14].

p53, an essential effector of the DNA damage response [17], [18] is also the most frequently inactivated tumor suppressor in human cancer [19], [20]. Through its transcriptional activity, p53 activates the expression of a number of genes important for diverse cellular processes including cell cycle arrest and apoptosis [17], [18]. p53 mutant mice have an increased risk of spontaneous tumor development, with most *p53^−/−^* mice succumbing by 6 months of age mainly due to lymphomas and sarcomas [21]. p53 deficiency also accelerates tumorigenesis in mutant mice lacking DNA damage repair proteins. For example, mice deficient for both p53 and either Xrcc4 or Lig4, two NHEJ factors, rapidly succumb to B-cell lymphomas that present chromosomal translocations resulting in c-Myc amplification [22], [23]. Similarly, loss of p53 also exacerbates tumorigenesis in mice lacking proteins important for homologous recombination (HR)-mediated repair such as Brca1 and Brca2 [17], [24], [25], [26], [27]. Furthermore, dual loss of p53 and the DSB signaling protein Rnf168 increases the frequency and shortens the latency of tumors [28].

In this study, we have generated *Rnf8^−/−^p53^−/−^* mice and examined the consequences of Rnf8 and p53 loss on development, DNA damage responses and cancer. We observed that loss of p53 reversed the increased radiosensitivity and premature senescence associated with the loss of Rnf8. Moreover, loss of p53 rescued the homeostasis defect observed in *Rnf8^−/−^* thymocytes and splenocytes, yet it failed to rescue the defects in the growth and CSR of *Rnf8^−/−^* mice. Concomitant loss of Rnf8 and p53 resulted in defects of both G1/S and G2/M checkpoints. Overall, loss of p53 potentiated genomic instability and dramatically accelerated tumor development in the absence of Rnf8, suggesting that an important functional interaction between p53 and Rnf8 exists, and is relevant for DDR and related mechanisms.

## Results

### *Rnf8^−/−^p53^−/−^* mice are viable

While Rnf8 plays important roles in DSB signaling and genomic integrity [3], [4], [5], [9], [14], p53 is known for its central role in the DDR as the “guardian of the genome” [29]. To examine functional interactions of Rnf8 and p53, we crossed *Rnf8^−/−^* mice (AS0574 strain) with *p53^−/−^* mice to ultimately generate *Rnf8^−/−^p53^−/−^* mice. These double mutant mice were born with a normal Mendelian ratio indicating that double-knockout of Rnf8 and p53 does not lead to embryonic lethality.

### p53 loss rescues some but not all of the growth defects of *Rnf8^−/−^* mice

*Rnf8^−/−^* mice have been previously shown to display growth defects as demonstrated by their smaller size compared to Wild-Type (*WT*) littermates [14]. Accordingly, we examined the consequence of p53 loss on the observed growth defects associated with Rnf8 deficiency. As expected, *Rnf8^−/−^* male mice weighed significantly less than their *WT* counterparts (22.8 g±1.4 vs. 32.1 g±2.5, P<0.0001; Figure S1A). *Rnf8^−/−^p53^−/−^* male mice were significantly smaller than their age matched *WT* littermates (20.6 g±1.3, P<0.0001), however they were no different in size compared to *Rnf8^−/−^* littermates (Figure S1A). A similar trend was observed for female mice where both *Rnf8^−/−^* and *Rnf8^−/−^p53^−/−^* weighted significantly less than matched *WT* female littermates (15.6 g±1.7 for *Rnf8^−/−^*, P = 0.0002 and 16 g±0.8 for *Rnf8^−/−^p53^−/−^*, P<0.0001 vs. 25 g±1.7 for *WT*; Figure S1B). No difference in weight was observed between *Rnf8^−/−^* and *Rnf8^−/−^p53^−/−^* females (Figure S1B). These data indicate that p53 loss does not rescue growth defects observed in *Rnf8^−/−^* mice.

**Figure 1:**
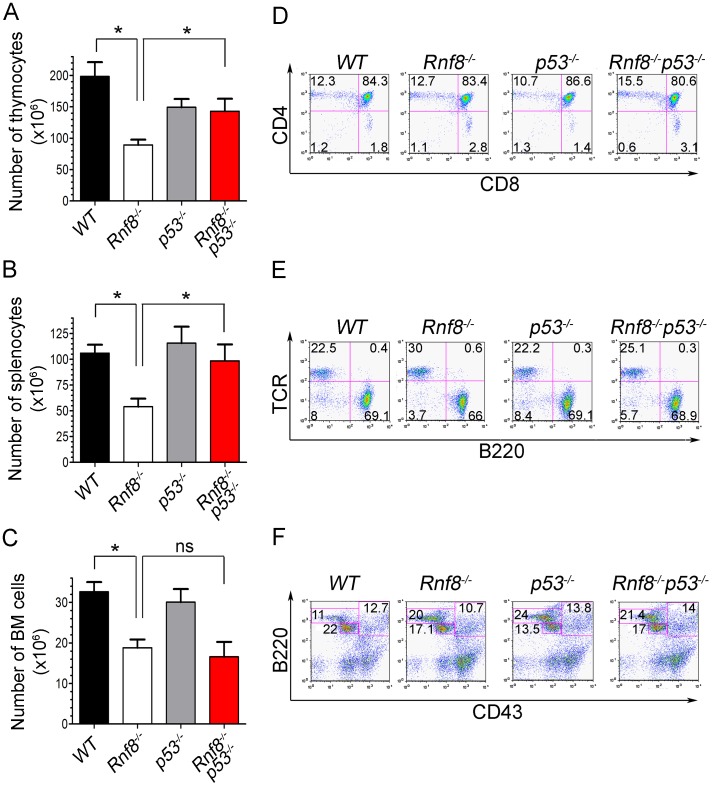
p53 loss rescues homeostasis of thymocytes and splenocytes, but not bone marrow populations deficient for Rnf8. (A) Thymus cell count in *Rnf8^−/−^p53^−/−^* mice. Total number of thymocytes was counted using trypan blue dye exclusion assay from 6-week old *Rnf8^−/−^p53^−/−^* and control mice. Data are presented as the means SD of at least 5 independent experiments. * represents P<0.05. Statistical analysis was performed using a student t-test. (B) Splenocyte cell count in *Rnf8^−/−^p53^−/−^* mice. Total number of cells was counted from freshly isolated spleen from 6-week-old *Rnf8^−/−^p53^−/−^* and control mice. Data are presented as the means SD of at least 5 independent experiments. * represents P<0.05. Statistical analysis was performed using a student t-test. (C) Bone marrow cell count in *Rnf8^−/−^p53^−/−^* mice. Cells isolated from one femur of 6-week old *Rnf8^−/−^p53^−/−^* mice and their controls were counted. Data are presented as the means SD of at least 5 independent experiments. Statistical analysis using student t-test indicated no significant difference between cells counts in *Rnf8^−/−^p53^−/−^* and *Rnf8^−/−^* mice. ns: Not significant. (D) Cell population profile in *Rnf8^−/−^p53^−/−^* thymus. Thymocytes from *Rnf8^−/−^p53^−/−^* and control 6 week-old mice were stained with anti-CD4 and anti-CD8 antibodies and the proportion of each thymocyte subpopulation was determined by flow cytometry. No difference was observed between Single positive (CD4^+^CD8^−^, CD4^−^CD8^+^), double positive (CD4^+^CD8^+^) or double negative (CD4^−^CD8^−^) subpopulation in *Rnf8^−/−^p53^−/−^* mice as compared to control mice. (E) Proportion of B- and T-cells in spleen from *Rnf8^−/−^p53^−/−^* mice and controls. Freshly isolated splenocytes from 6 week-old *Rnf8^−/−^p53^−/−^* mice and its controls were stained with anti-TCR and anti-B220 antibodies and the proportion of B-cells (B220^+^TCR^−^) and T-cells (B220^−^TCR^+^) were determined by flow cytometry. (F) *Rnf8^−/−^p53^−/−^* B-cell progenitor profile. Bone marrow cells from 6 week-old *Rnf8^−/−^p53^−/−^* and control mice were stained with anti-B220 and anti-CD43 antibodies and the frequency of each subpopulation was determined by flow cytometry.

*Rnf8* loss was also reported to result in reduced number of thymocytes, splenocytes and bone marrow cells [14], [16]. In order to investigate whether the impaired homeostasis of Rnf8-null immune cells was p53-dependent, we examined the numbers and subpopulations of splenocytes, thymocytes and bone marrow cells from 6-week-old *Rnf8^−/−^p53^−/−^*mice and their *Rnf8^−/−^*, *p53^−/−^* and *WT* littermates. Splenocyte, thymocyte and bone marrow cell numbers was significantly lower in *Rnf8^−/−^* mice compared to *WT* littermates (P = 0.0004, P = 0.001 and P = 0.0007 respectively; Figure 1A–1C). Interestingly, *Rnf8^−/−^p53^−/−^* mice and their *WT* and *p53^−/−^* littermates had similar numbers of splenocytes and thymocytes, indicating that loss of p53 rescues the reduced number of *Rnf8^−/−^* cells in these compartments (Figure 1A and 1B). In contrast, the reduced number of bone marrow cells in *Rnf8^−/−^* mice was also observed in *Rnf8^−/−^p53^−/−^* mice (Figure 1C). These data indicate that p53 loss rescues the number of thymocytes and splenocytes, but not bone marrow cells, in *Rnf8^−/−^* mice.

We next examined thymocytes, splenocytes and bone marrow cells by flow cytometry to characterize defects in immune cell subpopulations in *Rnf8^−/−^p53^−/−^* mice. The proportions of double positive (CD4^+^CD8^+^), single positive (CD4^+^ and CD8^+^), and T-cell receptor β (TCRβ) positive thymocytes were similar in all four genotypes (Figure 1D and Figure S2A). Representation of B- and T-cell populations in the spleen as well as proportions of recirculating (B220^high^CD43^−^IgM^+^), pre- (CD43^−^B220^+^IgM^−^) and pro-B (CD43^+^B220^+^IgM^−^) cells in the bone marrow were not affected by dual loss of Rnf8 and p53 (Figure 1E and 1F; and Figure S2A and S2B). Collectively these data illustrate that the impaired homeostasis of immune cells deficient for Rnf8 is dependent on p53 in the case of thymocytes and splenocytes but p53-independent in the case of bone marrow cells.

### Increased p53 and cleaved caspase-3 expression in *Rnf8^−/−^* thymus and spleen

**Figure 2:**
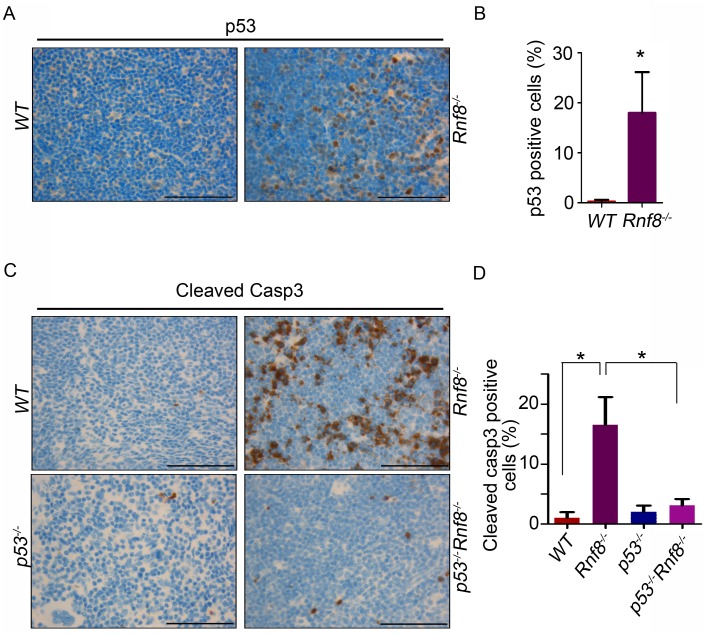
Increased p53 expression and cleaved caspase-3 levels in *Rnf8^−/^* ^−^ thymocytes. (A) p53 IHC staining of thymus sections of *Rnf8^−/−^* mice and *WT* littermates. (B) Quantification of p53 positive cells in thymus. An average of 20 randomly chosen fields was counted at 63× magnifications. (C) Cleaved caspase-3 levels in the thymus of *Rnf8^−/−^p53^−/−^* mice and control littermates. Anti-cleaved caspase-3 IHC staining of thymus of *Rnf8^−/−^p53^−/−^*, *Rnf8^−/−^*, *p53^−/−^* and *WT* mice. (D) Quantification of cleaved caspase-3 positive cells in the thymus. An average of 20 fields counted at 63× magnification. Data is representative of 3 different experiments. * indicates statistical significance (P<0.05). Bar: 50 µm.

Because loss of p53 rescued the reduced cell numbers in the thymus and spleen we wanted to determine whether p53-dependent apoptosis could be the cause behind the reduced cell numbers in these organs. Immunohistochemistry on tissues derived from *WT* and *Rnf8^−/−^* mice showed a tissue specific increase in p53 expression in *Rnf8^−/−^* mice. We observed a significant increase in the number p53-positive thymocytes in *Rnf8^−/−^* mice compared to *WT* littermates (18%±8.1 vs. 0.27%±0.3 respectively, P<0.0001; Figure 2A and 2B). Similarly, there was an increase in the number of p53-positive cells in the spleen of *Rnf8^−/−^* mice compared to *WT* littermates (17%±5.4 vs. 0.64%±0.49 respectively, P<0.0001; Figure S3A and S3B). In addition, immunohistochemistry was also performed on these tissues using cleaved caspase-3 antibody to detect apoptotic cells. We observed a dramatic increase in cleaved caspase-3 staining in the thymus of *Rnf8^−/−^* mice compared to *WT* controls (16.5%±4.6 for *Rnf8^−/−^* vs. 1%±0.9 for *WT*, P<0.0001; Figure 2C and 2D). However, the staining of cleaved caspase-3 was significantly reduced in the thymus of *Rnf8^−/−^p53^−/−^* mice compared to *Rnf8^−/−^* littermates (3.1%±1 vs. 16.5%±4.6, P<0.0001; Figure 2C and 2D). Similarly, levels of cleaved caspase-3 were also increased in *Rnf8^−/−^* splenocytes as compared to *WT* and *Rnf8^−/−^p53^−/−^* splenocytes (8.3%±3.5 for *Rnf8^−/−^* vs. 0.61%±0.49 for *WT*, P<0.0001 and 1.3%±0.99 for *Rnf8^−/−^p53^−/−^* P<0.0001; Figure S3C and S3D).

In order to determine if p53 is also activated in other *Rnf8^−/−^* tissues, we stained liver, kidney, lung, heart and intestine of *Rnf8^−/−^* mice and *WT* controls with anti-p53 antibodies. With the exception of the intestinal crypts of *Rnf8^−/−^* mice that showed increased frequency of p53 positive basal cells compared to *WT* controls (P<0.0001), p53 staining remained low and similar to *WT* mice in other tissues (Figure S4A and S4B).

These results suggest the activation of p53 in *Rnf8^−/−^* mice is tissue specific and that lower cellularity in the thymus and spleen of *Rnf8^−/−^* mice is caused by increased p53 activation and cell death in these tissues.

### *Rnf8^−/−^p53^−/−^* mice display class switch recombination defects

**Figure 3:**
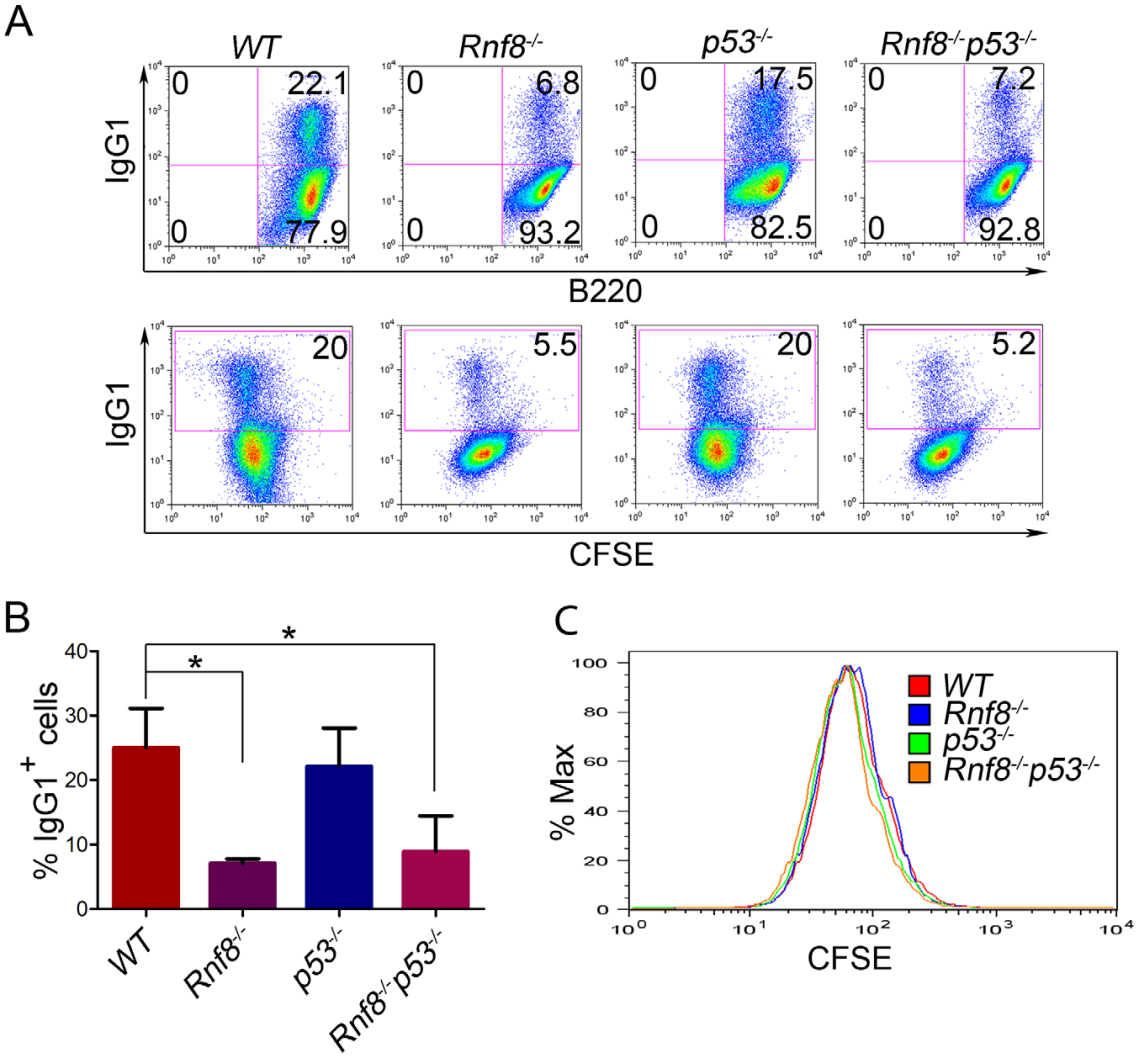
Loss of p53 does not rescue class switch recombination defects observed in *Rnf8^−/−^* mice. (A) B-cells were purified from the spleen of 6-week old *Rnf8^−/−^p53^−/−^* and control mice and class switch recombination was examined. B-cells were treated with anti-CD40 and IL-4 for 4 days, then the percentage of B-cells that have switched to IgG1 expression was determined by flow cytometry. Data presented are representative of at least 4 different experiments. (B) Quantification of Ig switching to IgG1. Percentage of B-cells that were IgG1^+^ after 4 days of stimulation *in vitro* in the presence of anti-CD40 and IL-4. Data are presented as the means SD of at least 4 independent experiments. * represents statistical significance (P<0.05) using a student t-test. (C) Cell division profiles of *Rnf8^−/−^p53^−/−^* B-cells and their controls. Cells were stained with CFSE and allowed to proliferate for 4 days in the presence of CD40 and Il-4. CFSE dilution profiles were examined using flow cytometry.

*Rnf8^−/−^* mice are immunodeficient and display CSR defects similar to what was observed with *H2ax^−/−^* and *Rnf168^−/−^* mice, yet less prominent than that observed in *53bp1^−/−^* mice [14], [16], [28], [30], [31], [32]. We examined whether p53 plays a role in the defective CSR associated with Rnf8 deficiency. The ability of B-cells purified from the spleens of *Rnf8^−/−^p53^−/−^* mice and littermate controls to undergo IgG1 CSR was examined in response to anti-CD40 and IL-4. *Rnf8^−/−^* B-cells presented a reduced ability to switch to IgG1 in response to anti-CD40 plus IL-4 compared to *WT* B-cells (7.1%±0.7 vs. 25%±6.1; P = 0.0004); and as reported [33], *p53^−/−^* B-cells showed similar level of CSR (22.1%±5.9) compared to *WT* controls (25%±6.1) (Figure 3A and 3B). Interestingly, the level of IgG1 CSR in *Rnf8^−/−^p53^−/−^* B-cells was significantly reduced compared to *WT* controls (8.9%±5.5 vs. 25%±6.1; P = 0.0029) and remained similar to that observed in *Rnf8^−/−^* B-cells (P>0.05; Figure 3A and 3B). This reduced ability to undergo class switch to IgG1 in the absence of Rnf8 was not caused by a defect in cell proliferation as B-cells of all four genotypes showed similar Carboxyfluorescein Diacetate Succinimidyl Ester (CFSE) profiles 96 hours following anti-CD40+IL4 stimulation (Figure 3C). Therefore, inactivation of p53 does not rescue or exacerbate CSR defects associated with the loss of Rnf8.

### Loss of p53 rescues radiosensitivity of *Rnf8^−/−^* mice and the growth defect of *Rnf8^−/−^* cells

**Figure 4:**
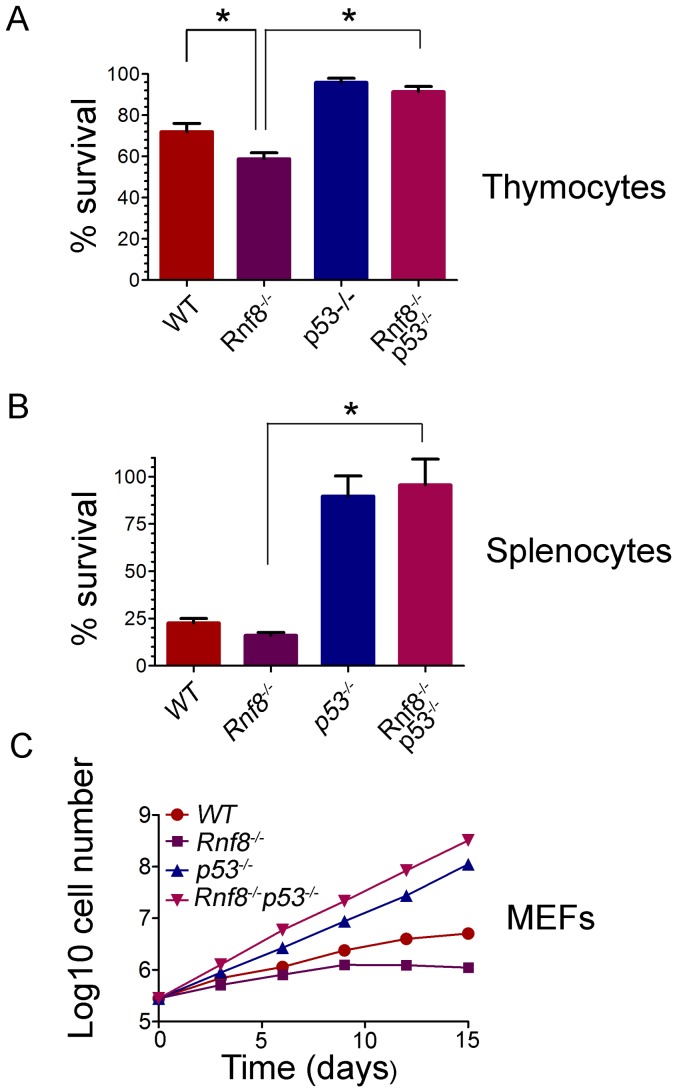
Radioresistance and increased proliferation of *Rnf8^−/−^* cells in the absence of p53. (A) Freshly isolated thymocytes from *Rnf8^−/−^p53^−/−^* mice and control littermates were irradiated with 2 Gy and cell death was determined 12 hours later. Data are presented as the means SD of at least 5 independent experiments. * represents significant difference (P<0.05; student t-test) compared to *WT* and *Rnf8^−/−^* controls. No difference was observed between *p53^−/−^* and *Rnf8^−/−^p53^−/−^* thymoctes. (B) Freshly isolated splenocytes were irradiated (2 Gy) and cell death was determined 24 hours later. Data are presented as the means SD of at least 5 independent experiments. * represents statistical significance (P<0.05; student t-test) compared to *WT* and *Rnf8^−/−^* controls. No significant difference was observed between *p53^−/−^* and *Rnf8^−/−^p53^−/−^* splenocytes. (C) Cumulative growth curve of *Rnf8^−/−^p53^−/−^* MEFs. Passage 2 *Rnf8^−/−^p53^−/−^* MEFs and controls were plated in 60 mm plates at a density of 3×10^5^ cells/plate. Cells were replated at the same density every 3 days and cumulative cell growth was calculated. Data are presented as the log10 of means ± SD of at least 5 independent experiments.

Our previous findings demonstrated increased radiosensitivity of *Rnf8^−/−^* thymocytes and bone marrow cells and sensitivity to whole body irradiation was also increased in *Rnf8^−/−^* mice compared to *WT* littermates [14]. In addition, p53 levels were increased in both untreated and gamma irradiated thymocytes from *Rnf8^−/−^* mice as compared to those of *WT* littermates [14]. In order to define whether the increased radiosensitivity of *Rnf8^−/−^* mice was dependent on p53, we examined cell death rates of *Rnf8^−/−^p53^−/−^*, *Rnf8^−/−^*, *p53^−/−^*, and *WT* thymocytes and splenocytes following IR-treatment. Freshly collected thymocytes from littermates with the four genotypes were subjected to IR (2 Gy) and cell death was examined 12 hours later using propidium iodide (PI) staining. As expected [14], *Rnf8^−/−^* thymocytes were significantly more radiosensitive compared to *WT* controls (P = 0.03; Figure 4A). However, in contrast to thymocytes from *Rnf8^−/−^* mice (P<0.0001) and *WT* mice (P = 0.034), but similar to *p53^−/−^* thymocytes (P>0.05), *Rnf8^−/−^p53^−/−^* thymocytes were highly radioresistant (Figure 4A). These results demonstrate that radiosensitivity of *Rnf8^−/−^* thymocytes was indeed p53-dependent.

We next examined responses to irradiation of splenocytes from *Rnf8^−/−^p53^−/−^* mice and littermates. Increased radiosensitivity was observed 24 hours post-irradiation (2 Gy) of *Rnf8^−/−^* splenocytes compared to splenocytes from *WT* littermates (P = 0.037, Figure 4B). However, in contrast to *Rnf8^−/−^* and *WT* splenocytes (P<0.0001), and similar to *p53^−/−^* splenocytes (P>0.05), *Rnf8^−/−^p53^−/−^* splenocytes were completely radioresistant (Figure 4B). These data suggest that like thymocytes, increased irradiation-induced cell death of *Rnf8^−/−^* splenocytes was p53-dependent.

It has been reported that *Rnf8^−/−^* mouse embryonic fibroblasts (MEFs) exhibited growth defects [14]. Thus, we addressed the effect of p53 on the growth defect of *Rnf8^−/−^* MEFs by performing 3T3 passaging of *Rnf8^−/−^p53^−/−^* MEFs and their *Rnf8^−/−^*, *p53^−/−^* and *WT* controls. We found that *Rnf8^−/−^* MEFs grew slower than *WT* MEFs, However, loss of p53 completely rescued this growth defect such that *Rnf8^−/−^p53^−/−^* MEFs grew significantly faster than *Rnf8^−/−^* MEFs (P = 0.0006 at day 3, P = 0.0015 at day 6, P = 0.0015 at day 9, P = 0.0118 at day 12 and P = 0.0195 at day 15; Figure 4C and Table S1). The growth rate of *Rnf8^−/−^p53^−/−^* MEFs was similar to that of *p53^−/−^* mutant controls (Figure 4C and Table S1).

### Senescence of *Rnf8^−/−^* MEFs is p53-dependent

**Figure 5:**
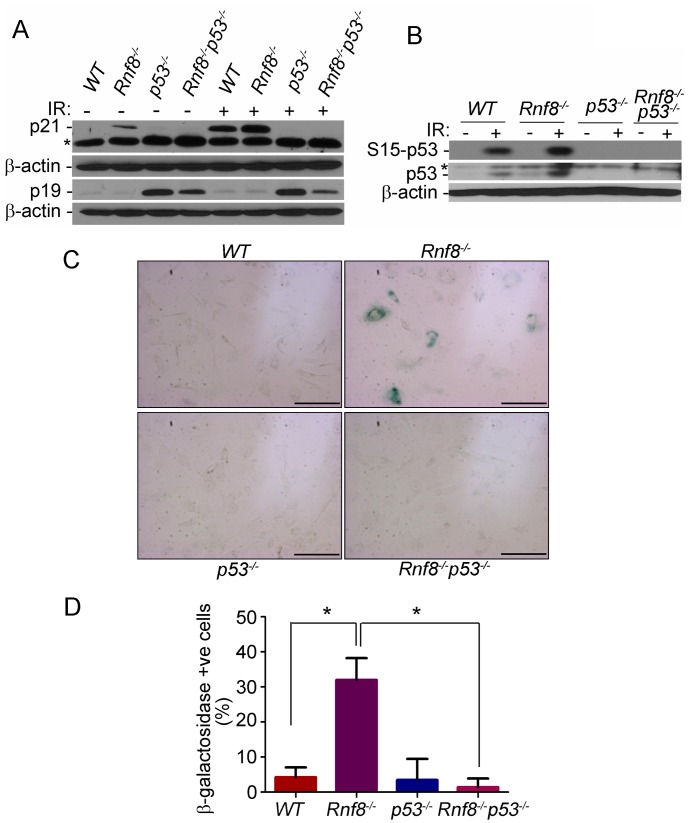
p53 deficiency rescues the senescence of *Rnf8^−/−^* MEFs. (A) Western blot analysis of *Rnf8^−/−^p53^−/−^* and control MEFs for p21 and p19^ARF^ under untreated conditions and following 8 Gy ionizing radiation. β-actin is used as a loading control. * indicates a non specific band. (B) Western blot analysis of phosphorylated Ser-p53 and total p53 in untreated or γ-irradiated *Rnf8^−/−^p53^−/−^* and control MEFs. β-actin was used as a loading control. * indicates a non specific band. (C) Senescence-associated β-galactosidase staining of *Rnf8^−/−^* MEFs and controls. MEFs were fixed and stained with X-gal solutions. The cells were then visualized using a brightfield microscope. These images are representative of three different experiments. (D) Quantification of the percentages of β-galactosidase-positive cells for 3 different experiments. * indicates P<0.05. Bar: 500 µm.

Because *Rnf8^−/−^* MEFs display severe growth defects that are completely abrogated in the absence of p53 we wanted to test whether these cells undergo p53-induced senescence. Primary MEFs can undergo a limited number of replications before they enter a senescence state. Senescence in MEFs is usually associated with increased levels of p53 and its downstream target p21 as well as increased levels of p19^ARF^ [34]. We therefore examined levels of p21, p19^ARF^ and p53 under both untreated conditions and following γ-irradiation in passage 3 MEFs. We found that p21 levels were higher in *Rnf8^−/−^* MEFs compared to *WT*, *p53^−/−^* and *Rnf8^−/−^p53^−/−^* MEFs under both basal and irradiated conditions (Figure 5A). In contrast, levels of p19^ARF^ were similar and very low in both *WT* and *Rnf8^−/−^* MEFs (Figure 5A). Consistent with previous studies of *p53^−/−^* MEFs [35], levels of p19^ARF^ were elevated in both *p53^−/−^* and *Rnf8^−/−^p53^−/−^* MEFs, (Figure 5A). Examination of p53 indicated increased levels of total p53 and its phosphorylated Ser-15 form in *Rnf8^−/−^* MEFs compared to *WT* controls under both untreated and irradiated conditions (Figure 5B). As p21 is a transcriptional target of p53, its increased expression levels in *Rnf8^−/−^* MEFs is most likely caused by the increased activation of p53 in these cells (Figure 5A and 5B).

Increased levels of senescence associated β-galactosidase have been used as a biomarker for senescence [34]. We therefore used this method to determine whether the reduced growth in *Rnf8^−/−^* MEFs was caused by senescence. We found that there was a significantly higher percentage of *Rnf8^−/−^* MEFs that stained positive for the senescence associated β-galactosidase compared to *WT* (P = 0.0022), *p53^−/−^* (P = 0.0047) and *Rnf8^−/−^p53^−/−^* (P<0.0001) MEFs respectively (Figure 5C and 5D). These results point to increased senescence of *Rnf8^−/−^* MEFs in a manner dependent on p53 and p21 but independent of p19^ARF^.

### *Rnf8^−/−^p53^−/−^* MEFs display both G1/S and G2/M DNA damage checkpoint defects

**Figure 6:**
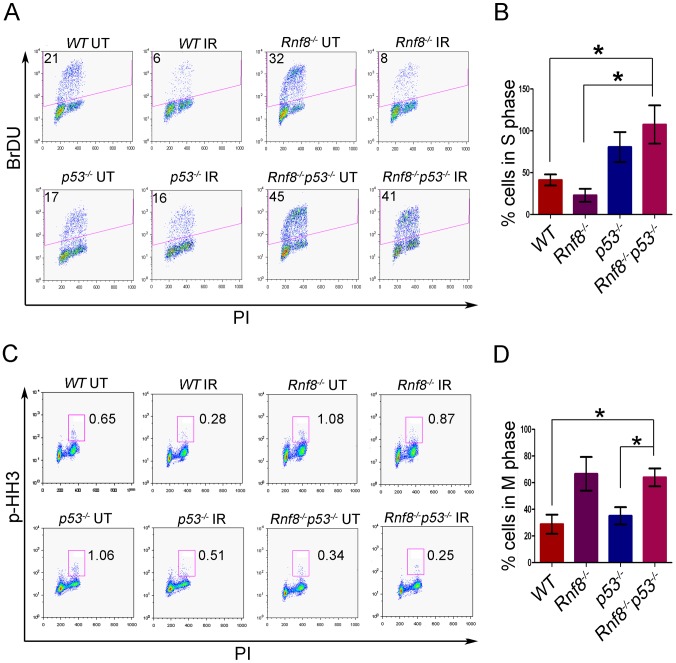
Loss of Rnf8 and p53 results in defective cell cycle checkpoints. (A) G1-S checkpoint analysis. *Rnf8^−/−^p53^−/−^* early passage primary MEFs and their controls were left untreated or irradiated with 10 Gy and 18 hours later, cells were pulsed with BrDU for 4 hours. The percentage of cells in S-phase was determined by flow cytometry. A representative of 3 independent experiments is shown. (B) Quantification of the percentage of cells remaining in S phase after irradiation relative to untreated controls. Data are presented as the means SD of at least 3 independent experiments. * denotes statistical significance (P<0.05, student t-test). There is no statistical difference between *p53^−/−^* and *Rnf8^−/−^p53^−/−^* MEFs. (C) G2-M checkpoint analysis. *Rnf8^−/−^p53^−/−^* early passage primary MEFs and their controls were irradiated with 2 Gy or left untreated, then allowed to recover for 1 hour. Cells in M phase were determined by staining with PI and p-HH3. A representative of 3 independent experiments is shown. (D) Quantification of the percentage of cells in M phase following irradiation relative to untreated. Data are presented as the means SD of at least 3 independent experiments. * denotes statistical significance (P<0.05; student t-test).

Cell cycle checkpoints are activated in response to DSBs, thereby preventing cells from progressing to the next phase of the cell cycle before the repair of damaged DNA. The two main cell cycle checkpoints are the G1/S and the G2/M checkpoints [36]. While p53 plays roles in both G1/S and G2/M checkpoints [36], [37], defective G2/M checkpoint activation has been observed in Rnf8 deficient cells [5], [38]. We examined the effect of combined loss of Rnf8 and p53 on cell cycle checkpoints following IR-induced DNA damage. Our data indicated that only 41.3%±3.8% of *WT* MEFs and 23%±4.5% of *Rnf8^−/−^* MEFs were in the S phase following treatment with IR (10 Gy) as compared to untreated MEFs (Figure 6A and 6B). These results suggest that Rnf8 is dispensable for the execution of the G1/S checkpoint. Our data also indicated that in contrast to *WT* and *Rnf8^−/−^* MEFs, 80.7%±10.3% of *p53^−/−^* MEFs and 107%±13.2% of *Rnf8^−/−^p53^−/−^* MEFs were in the S phase of the cell cycle 22 hours post exposure to 10 Gy of irradiation (Figure 6A and 6B). The percentage of cells in S phase post-irradiation compared to untreated controls was significantly higher in *Rnf8^−/−^p53^−/−^* MEFs compared to both *WT* and *Rnf8^−/−^* MEFs (P = 0.0085 and P = 0.0037 respectively; Figure 6B).

We next examined the consequence of p53 loss on G2/M checkpoint defects associated with Rnf8 deficiency [5], [38]. *Rnf8^−/−^p53^−/−^* MEFs and their controls were either untreated or irradiated (2 Gy) and their activation of G2/M checkpoint was examined 1 hour later. Combined loss of Rnf8 and p53 resulted in G2/M checkpoint defects similar to *Rnf8^−/−^* MEFs (P>0.05; Figure 6C and 6D). The percentage of irradiated cells in mitosis compared to untreated controls was significantly higher in *Rnf8^−/−^p53^−/−^* MEFs (64%±6.7%) compared to *WT* (28.9%±7.2%; P = 0.0235) and *p53^−/−^* MEFs (35.2±6.5; P = 0.0367); but it remained similar to *Rnf8^−/−^* MEFs (66.7%±12.6%). Therefore, *Rnf8^−/−^p53^−/−^* MEFs displayed both the G1/S and G2/M checkpoint defects observed in *p53^−/−^* and *Rnf8^−/−^* single mutant MEFs respectively.

### *Rnf8^−/−^p53^−/−^* MEFs display increased spontaneous and residual γ-H2ax and Mdc1 foci but have defective 53bp1 and Brca1 foci formation

*Rnf8^−/−^* MEFs were reported to display transient, but not sustained, Irradiation Induced Foci (IRIF) for 53bp1 indicating a defect in DSB signaling [14], [39]. We examined the effect of p53 loss on the ability of *Rnf8^−/−^* MEFs to recruit and retain 53bp1 at DSB flanking sites. Similar to *Rnf8^−/−^* MEFs, and in contrast to *p53^−/−^* and *WT* MEFs, *Rnf8^−/−^p53^−/−^* MEFs displayed only small transient 53bp1 foci at early time point (0.5 hour) post-IR (8 Gy) and were unable to retain 53bp1 proteins at DSB sites (Figure S5).

**Figure 7:**
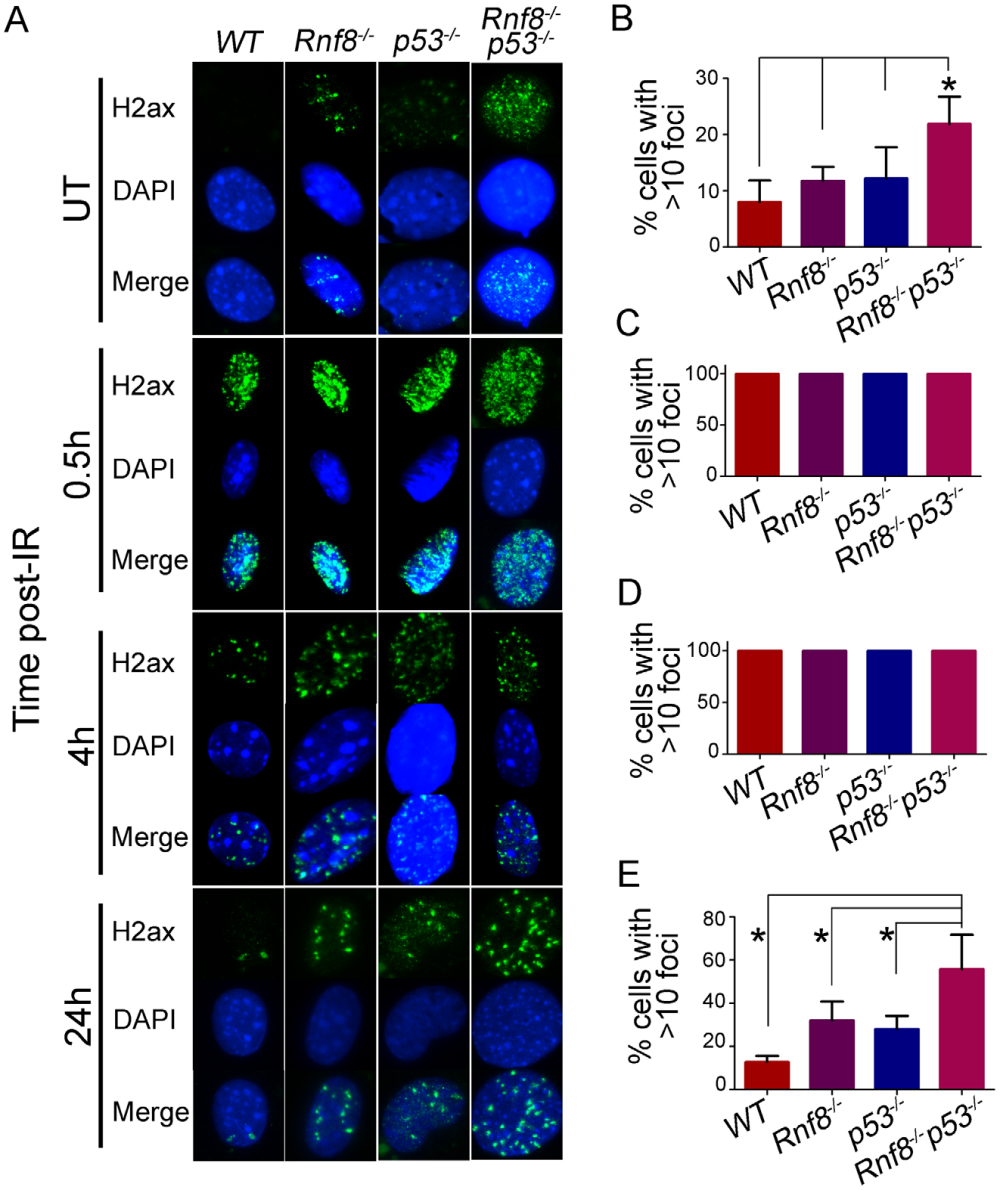
Increased basal and residual γ-H2ax foci in *Rnf8^−/−^p53^−/−^* MEFs. (A) γ-H2ax staining for *Rnf8^−/−^p53^−/−^* MEFs and controls. *Rnf8^−/−^p53^−/−^*, *Rnf8^−/−^*, *p53^−/−^* and *WT* early passage primary MEFs were either mock-treated (UT) or irradiated (8 Gy) and allowed to recover for 0.5, 4 and 24 hours. Cells were then fixed, stained using anti-γ-H2ax antibody and counterstained with DAPI. (B) Percentage of untreated cells that contained 10 or more γ-H2ax foci. Data are presented as the means SD of at least 3 independent experiments. At least 100 cells were quantified per experiment. * denotes statistical significance (P<0.05; student t-test). (C, D) Percentage of cells that showed more than 10 γ-H2ax foci at 0.5 and 4 hours post-irradiation respectively. At least 100 cells were quantified for each experiment. (E) Percentage of cells showing 10 or more γ-H2ax foci 24 hours after irradiation. Data are presented as the means SD of at least 3 independent experiments. At least 100 cells were quantified per experiment. * indicates statistical significance (P<0.05; student t-test).

Cell cycle checkpoints are crucial for preventing accumulation of damaged DNA in cells [36]. Because *Rnf8^−/−^p53^−/−^* MEFs have defective cell cycle checkpoints and defective DSB signaling as indicated by the impaired recruitment of 53bp1 to DSB sites, we examined whether these double mutant cells might also exhibit increased spontaneous DSBs compared to *Rnf8^−/−^*, *p53^−/−^* and *WT* control MEFs. In response to DSBs, γ-H2ax accumulates at the sites of the breaks and forms distinct nuclear foci that can be used to quantify DSBs [1]. Examination of γ-H2ax foci indicated that untreated *Rnf8^−/−^p53^−/−^* MEFs had significantly higher frequency of spontaneous γ-H2ax foci compared to *Rnf8^−/−^* (P = 0.0117), *p53^−/−^* (P = 0.0185) and *WT* (P = 0.0048) MEFs (Figure 7A and 7B).

No differences were observed between the four genotypes when cells were examined at early time points (0.5 and 4 hours) post-IR (8 Gy) for the presence of 10 or more γ-H2ax foci. In contrast, at the 24 hours time point post-IR, where most DSBs have been repaired in *WT* cells, the percentage of *Rnf8^−/−^p53^−/−^* MEFs that displayed 10 or more γ-H2ax foci was significantly higher compared to *Rnf8^−/−^* (P = 0.0403), *p53^−/−^* (P = 0.0175) and *WT* (P = 0.0018) MEFs (Figure 7A and 7C–7E).

We next examined Mdc1 foci as its phosphorylation is required for binding of Rnf8 to DSBs [5]. Similar to what we have observed with γ-H2ax, *Rnf8^−/−^p53^−/−^* MEFs displayed higher numbers of Mdc1 foci under both untreated conditions and during later time points following γ-irradiation (Figure S6). The increased number of *Rnf8^−/−^p53^−/−^* MEFs with more than 10 Mdc1 foci was statistically significant compared to *WT* (P = 0.0026), *Rnf8^−/−^* (P = 0.042) and *p53^−/−^* (P = 0.002) under untreated conditions (Figure S6). 24 hrs following irradiation, we also observed increased number of *Rnf8^−/−^p53^−/−^* MEFs displaying more than 10 residual Mdc1 foci compared to *WT* (P = 0.0006), *Rnf8^−/−^* (P = 0.0031) and *p53^−/−^* (P = 0.0008) (Figure S6). The same number of foci was observed in all genotypes 30 minutes following irradiation (Figure S6).

In addition to 53bp1, loss of Rnf8 also impairs the recruitment of Brca1 to the sites of DSBs [5]. Brca1 is involved in cell cycle checkpoint and in the promotion of DSB repair through homologous recombination [40], [41]. We found that there was a significant decrease in the number of MEFs with more that 10 Brca1 foci in *Rnf8^−/−^* and *Rnf8^−/−^p53^−/−^* backgrounds compared to *WT* MEFs at 6 and 24 hrs post-irradiation (P<0.0001) (Figure S7).

Collectively, the increased foci formation of γ-H2ax and Mdc1, that function upstream of Rnf8 in DSB, and the defective recruitment and retention at DSB sites of the downstream cell cycle checkpoint and DNA repair proteins 53bp1 and Brca1 respectively suggest defective DSB signaling and repair in the *Rnf8^−/−^p53^−/−^* MEFs.

### Dual loss of Rnf8 and p53 leads to increased levels of genomic instability

**Figure 8:**
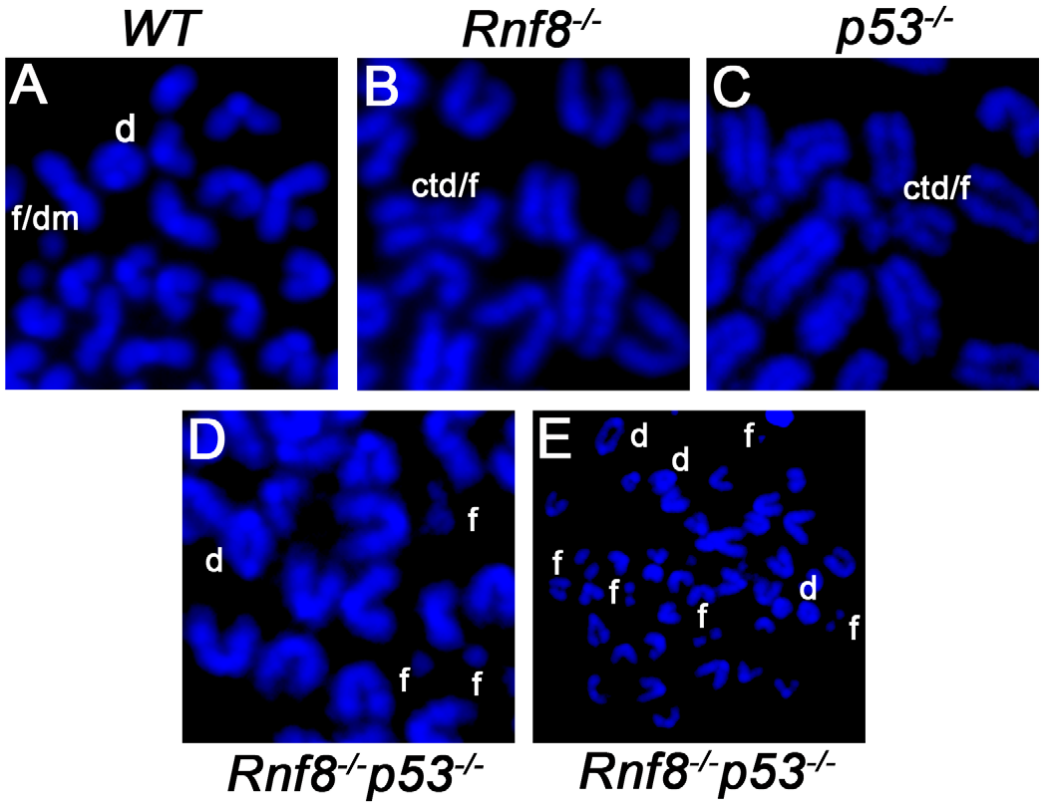
Representative chromosomal abnormalities observed in activated B-cells from *Rnf8^−/−^p53^−/−^* mice and control littermates. Representative metaphase spreads of irradiated (2 Gy) *WT* (A), *Rnf8^−/−^* (B), *p53^−/−^* (C) and *Rnf8^−/−^p53^−/−^* (D and E) LPS-activated B-cells. d: dicentric chromosome, f: chromosome fragments, dm: double minute chromosomes, ctd: chromatid type exchange/breaks.

Defects in DSB signaling or cell cycle checkpoints have been associated with increased genomic instability [42]. We previously reported increased genomic instability in B-cells lacking Rnf8 [14]. In addition, due to its major functions in DDR, loss of p53 also impairs genomic integrity [29]. To examine the effect of dual loss of Rnf8 and p53 on genomic integrity, we examined metaphase spreads from untreated and irradiated (2 Gy) LPS-activated B-cells from *Rnf8^−/−^p53^−/−^* mice and their *Rnf8^−/−^*, *p53^−/−^* and *WT* littermate controls. Under untreated conditions, the frequency of total aberrations in *Rnf8^−/−^p53^−/−^* B-cells was significantly higher compared to *WT* (P = 0.001), *Rnf8^−/−^* (P = 0.021) and *p53^−/−^* (P = 0.004) B-cells (Table 1). In response to IR (2 Gy), total aberrations were significantly higher in *Rnf8^−/−^p53^−/−^* B-cells compared to *WT* (P = 1.5×10^−6^), *Rnf8^−/−^* (P = 0.0025) and *p53^−/−^* (P = 8.6×10^−6^) (Table 1 and Figure 8). These data were consistent with the radioresistance and the defects in DSB signaling and checkpoints activation observed in *Rnf8^−/−^p53^−/−^* cells, and suggested that Rnf8 and p53 cooperate to maintain genomic integrity.

**Table 1:** Dual loss of Rnf8 and p53 leads to increased aneuploidy and genomic instability.

Genotypes/treatment	Metaphases scored	Aneuploid cells	Aberrant cells	Fragments/breaks	Fusions	Total aberrations
*WT*UT	113	0**0.00**	0**0.00**	1**0.88**	0**0.00**	1**0.88**
*Rnf8^−/−^*UT	163	8**4.91**	5**3.07**	6**3.68**	0**0.00**	6**3.68**
*p53^−/−^*UT	118	8**6.78**	3**2.54**	2**1.7**	0**0.00**	2**1.7**
*Rnf8^−/−^p53^−/−^*UT	183	30**16.4**	16**8.74**	18**9.83**	1**0.54**	19**10.4**
*WT*2 Gy	103	6**5.82**	13**12.6**	10**9.70**	4**3.88**	14**13.6**
*Rnf8^−/−^*2 Gy	104	13**12.5**	16**15.4**	16**15.4**	9**8.65**	25**24.0**
*p53^−/−^*2 Gy	104	5**4.81**	11**10.6**	13**12.5**	3**2.88**	16**15.4**
*Rnf8^−/−^p53^−/−^*2 Gy	105	15**14.2**	21**20.0**	34**32.4**	12**11.4**	46**43.8**

Splenocytes isolated from 6-week old *Rnf8^−/−^p53^−/−^* mice and control littermates were activated with LPS for 24 hours and then either left untreated (UT) or irradiated (2 Gy). 24 hours later metaphase spreads were prepared. Results shown are pooled from analyses of metaphase spreads from activated B-cells of at least two different mice per genotype. Second row (bold) - percentage data shown. Fusions involve dicentric chromosomes, sister chromatid fusion, ring chromosomes and Robertsonian fusion like configurations.

### Rnf8 cooperates with p53 in tumor suppression

**Figure 9:**
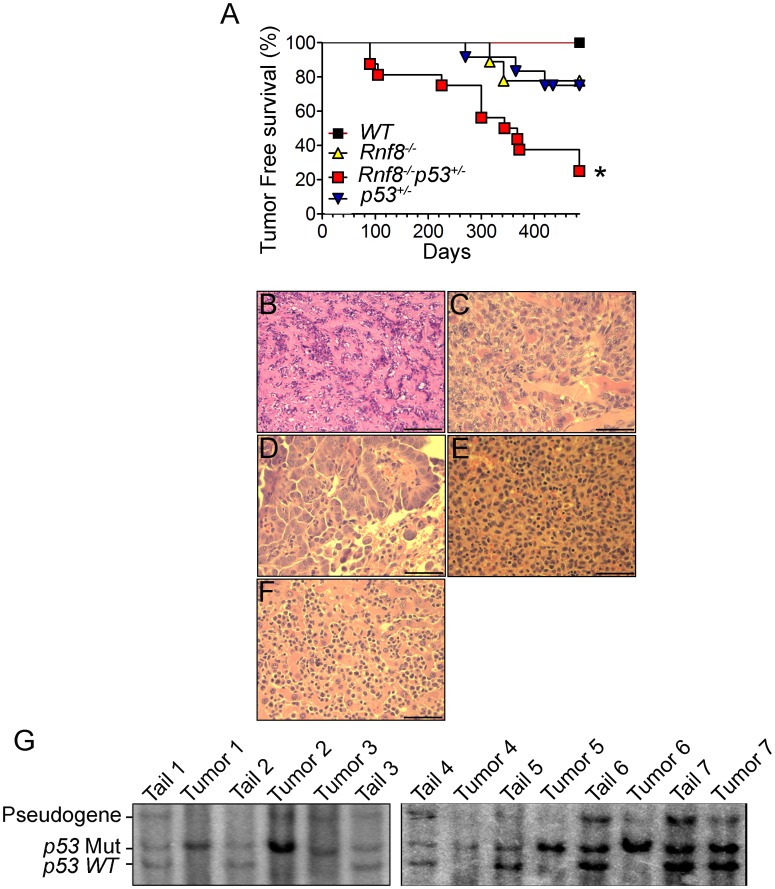
Increased tumor incidence and modification of the tumor spectrum in *Rnf8^−/−^p53^+/−^* mice. (A) Kaplan-Meir tumor free survival curve for *Rnf8^−/−^p53^+/−^* mice and control littermates. Survival of *Rnf8^−/−^p53^+/−^* mice (n = 15) is significantly decreased compared to *Rnf8^−/−^* (n = 9), *p53^+/−^* (n = 12) and *WT* (n = 7) littermates (P<0.05; Log rank test). (B) Osteoid osteoma that is composed of extracellular matrix resembling osteoid interspersed with groups of osteoblastic cells. These cells do not display any cytological features suggesting malignancy. The tumor is very homogeneous in nature, well vascularized and lacking any necrotic foci. (C) Rhabdomyosarcoma showing good vascularization, a high mitotic index and the absence of necrosis. (D) Lung adenocarcinoma compressing adjacent alveoli (lower right corner). (E) Spleen tissue that has been completely invaded by lymphoma. (F) The same lymphoma has also invaded liver tissue. Bars: B: 200 µm, C: 100 µm, D–G: 50 µm. (G) Southern blot showing loss of *WT* p53 allele in *Rnf8^−/−^p53^+/−^* tumors (tumor 1: rhabdomyosarcoma; tumors 2 & 3: thymomas, tumor 4 & 5 lymphomas, tumors 6 & 7 thymomas). Tail DNA of the same mouse was used as a control.

p53 is the most frequently inactivated tumor suppressor in human cancer [18]. Recent data has indicated that Rnf8 also functions as a tumor suppressor, and that about 40% of Rnf8-deficient mice develop tumors, mostly thymic and B-cell lymphomas [14]. To examine the functional interactions of p53 and Rnf8 in suppressing cancer; we first monitored cohorts of *Rnf8^−/−^p53^+/−^*, *Rnf8^−/−^* ¸ *p53^+/−^* and *WT* mice for one and a half years for tumor development and Kaplan-Meier tumor free survival analysis was performed. Tumor-free survival of *Rnf8^−/−^p53^+/−^* mice was significantly reduced compared to *WT* (P = 0.0029), *p53^+/−^* (P = 0.0115) and *Rnf8^−/−^* (P = 0.0188) controls (Figure 9A). *Rnf8^−/−^p53^+/−^* mice developed a wide range of tumors. 20% of the *Rnf8^−/−^p53^+/−^* mice (3 out of 15) developed hindlimb paralysis and in one of these mice the cause of the hindlimb paralysis was determined to be a benign osteiod osteoma in the spinal cord (Figure 9B). In addition, 7% of *Rnf8^−/−^p53^+/−^* mice (1 out of 15) developed rhabdomyosarcoma (Figure 9C) and lung adenocarcinoma (Figure 9D). By 485 days, 40% of the *Rnf8^−/−^p53^+/−^* mice had developed invasive lymphomas that disseminated into organs including the lung, spleen and liver (Figure 9E and 9F). Therefore, loss of one allele of p53 in the absence of Rnf8 can accelerate tumorigenesis without significantly affecting the tumor spectrum of *Rnf8^−/−^* mice (which develop mainly lymphomas) or *p53^+/−^* mice (which develop primarily sarcomas and lymphomas) [14], [43].

It has been previously reported that less than 50% of tumors that spontaneously develop in *p53^+/−^* mice exhibit loss of heterozygocity (LOH) [44]. In addition, the level of p53 LOH in tumors from *p53^+/−^* mice exposed to mutagenic carcinogens was found to vary depending on the tumor types [45]. We therefore used Southern blot analysis to determine the status of the *WT* p53 allele in 8 different tumors (4 thymic lymphomas, 1 rhabdomyosarcoma and 3 B-cell lymphomas) derived from *Rnf8^−/−^p53^+/−^* mice. Our data indicated that the *WT* p53 allele was lost in the majority (75%) of the *Rnf8^−/−^p53^+/−^* tumors examined (Figure 9G). These data suggest a selective pressure for the loss of the remaining *WT* p53 allele is behind the accelerated tumor development in *Rnf8^−/−^p53^+/−^* mice.

**Figure 10:**
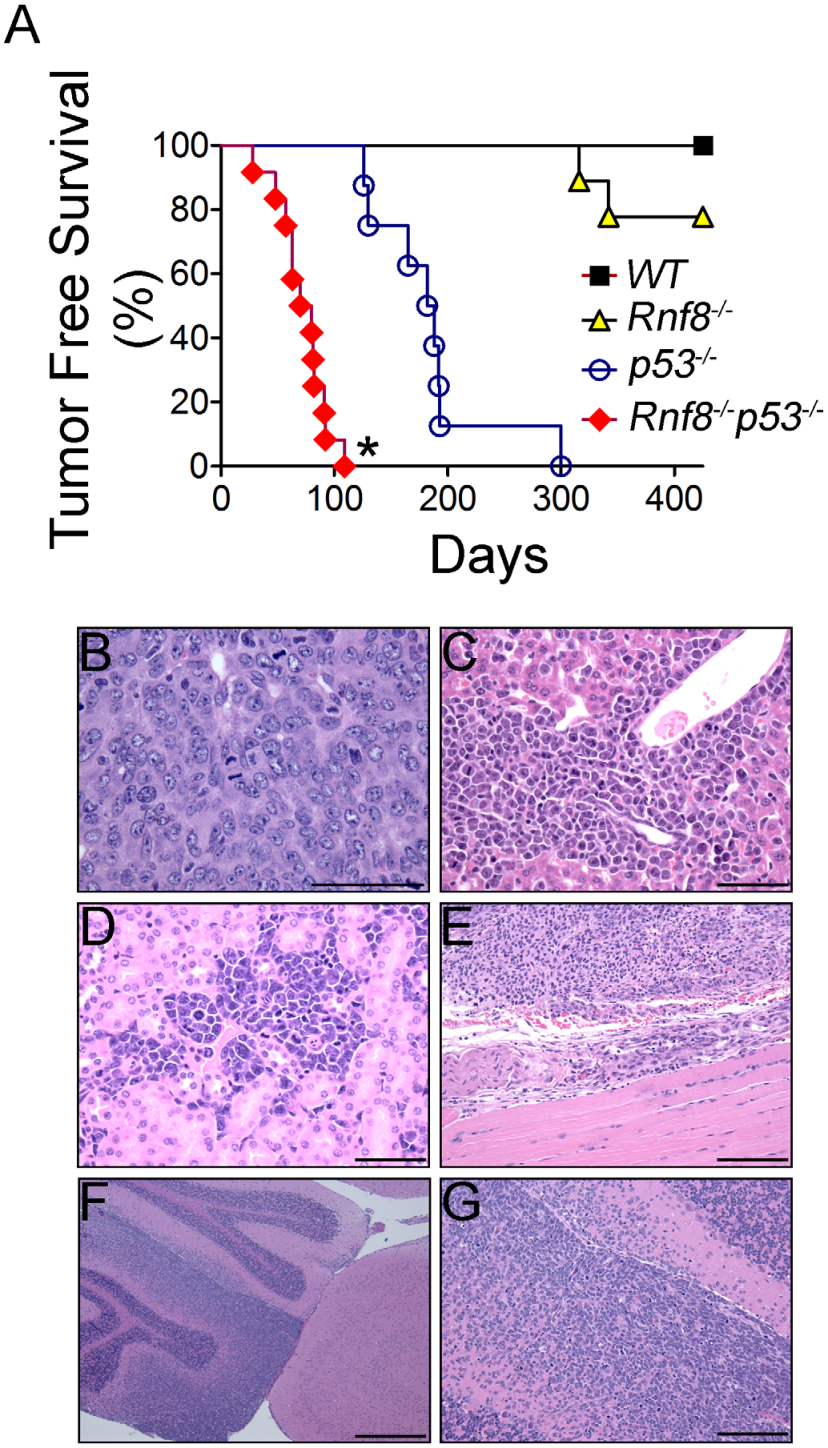
Cooperation of Rnf8 and p53 in the prevention of tumorigenesis. (A) Kaplan-Meier tumor free survival analysis of cohorts of *Rnf8^−/−^p53^−/−^* (n = 12), *Rnf8^−/−^* (n = 9), *p53^−/−^* (n = 9), and *WT* (n = 7) mice. Mice were monitored for one year and their tumor free survival curves were established. * indicates statistical significance compared to *p53^−/−^* and *Rnf8^−/−^* mice (P<0.05; log-rank test). (B) H&E staining of *Rnf8^−/−^p53^−/−^* thymoma. This tumor is composed of large and pleomorphic lymphocytes displaying sparse chromatin, prominent nucleoli and high mitotic index. (C) *Rnf8^−/−^p53^−/−^* lymphoma invading the liver. Liver infiltration is particularly evident around periportal areas. (D) *Rnf8^−/−^p53^−/−^* lymphoma invading the kidneys. Multiple tumor infiltrates are present in the interstitial peritubular spaces and display very high mitotic activity. (E) *Rnf8^−/−^p53^−/−^* lymphoma invading skeletal muscle tissue. (F) *Rnf8^−/−^p53^−/−^* lymphoma infiltrating the cerebellum. This figure shows a normal cerebellar cortex adjacent to cortex that has been mostly infiltrated by lymphoma. (G) Compromised cerebellum infiltrated by *Rnf8^−/−^p53^−/−^* lymphoma. Bars: B–D 50 µm, E and G 100 µm and F 500 µm.

*p53^−/−^* mice are highly prone to development of spontaneous tumors which are mostly thymomas. These mice have a median tumor incidence of about 4.5 months and they all succumb to tumors by the age of 10 months [21]. To examine the effect of dual loss Rnf8 and p53 on tumor development, we monitored cohorts of *Rnf8^−/−^p53^−/−^*mice and their *Rnf8^−/−^* ¸ *p53^−/−^* and *WT* controls for one year, and Kaplan-Meier tumor free survival analysis was performed. While as expected tumors were observed in *Rnf8^−/−^* and *p53^−/−^* mice, double mutants displayed higher cancer risk compared to single mutant littermates (Log-rank test, P<0.0001 compared to *Rnf8^−/−^* mice and P = 0.0004 compared to *p53^−/−^* mice; Figure 10A). Double mutant mice developed tumors with a remarkably short latency compared to single mutants. While the median tumor free survival was 185 days for our *p53^−/−^* cohort of mice, it was significantly reduced to 80 days for *Rnf8^−/−^p53^−/−^* mice. By 12 weeks of age all of *Rnf8^−/−^p53^−/−^* mice had succumbed to thymic lymphomas and/or B-cell lymphomas. One *Rnf8^−/−^p53^−/−^* mouse in the survival cohort developed both a B-cell lymphoma (B220^+^IgM^+^) (Figure S8A) and a thymic lymphoma (CD4^+^CD8^+^) (Figure S8B), whereas the rest of the cohort developed thymic lymphomas exclusively (Figure S8C). These lymphomas had very high mitotic index (Figure 10B), and were able to invade the liver (Figure 10C), kidney (Figure 10D), and skeletal muscle tissues (Figure 10E). Histopathological analysis of two *Rnf8^−/−^p53^−/−^* mice that had developed ataxia and an unsteady gait indicated that these mice suffered lymphoma that had crossed the meninges and disseminated into the cerebellum and the cerebrum which could explain the observed neurological defects (Figure 10F and 10G). Collectively, these data indicate that dual loss of Rnf8 and p53 exacerbated the risks for cancer development.

## Discussion

Post-translational modification by ubiquitin plays a critical role in the signaling and repair of DSBs [1], [2], [46]. In response to these DNA breaks, the E3 ubiquitin ligase Rnf8 ubiquitylates chromatin components H2a, H2ax and H2b at the flanking regions of DSBs [3], [4], [5], [6]. This ubiquitylation facilitates the recruitment to DNA break sites of a number of DNA damage response proteins (e.g. 53bp1 and Brca1) which are required for the activation of cell cycle checkpoints and effective DNA damage repair [2], [10].

Loss of Rnf8 in mice results in many defects including reduced growth, radiosensitivity, male sterility and immunodeficiency [14], [15], [16]. We have previously reported increased levels of p53 expression and activation in untreated and irradiated *Rnf8^−/−^* cells compared to *WT* controls [14]. We hypothesized that elevated levels of p53 occurs in response to defective repair of DNA damage in Rnf8 deficient cells and that this is the underlying cause for the increased radiosensitivity and the growth defects observed in *Rnf8^−/−^* mice.

p53 is crucial for the response to DSBs as it eliminates cells unable to repair damaged DNA, thus preventing increased genomic instability and suppressing cancer development [20]. Due to its important functions, loss of p53 has been reported to fully or partially rescue the growth and the embryonic and postnatal developmental defects associated with impaired DNA damage signaling and repair. For example, mice deficient for Brca1, Xrcc4 or Lig4 die during embryonic development, and loss of p53 either prolongs their embryonic development in the case of *Brca1* null mutants [47] or fully restores the viability of these mice in the case of *Xrcc4* and *Lig4* mutants [22], [23].

Our data indicate that loss of p53 completely restrained the elevated radiosensitivity of *Rnf8^−/−^* thymus and spleen, reflecting a complete dependence of this radiosensitivity on the p53 pathway. In these two organs specifically, p53 and cleaved caspase-3 levels were elevated, which might indicate that p53 and caspase-3-dependent apoptosis occurs in these organs but not in others such as heart, lung, kidney and liver. Interestingly, basal cells of the intestinal crypts are the only non-hematopoietic cells that displayed increased p53 levels in the absence of Rnf8. It is possible that *Rnf8^−/−^* cell types that do not generate programmed DSBs during development or that are less prone to generate spontaneous DSBs due to low rate of proliferation associated stalled replication would not display increased p53 levels.

Rnf8 is required for efficient recruitment of Brca1 to DSB flanking sites [4], [9], and our data indicate that as in *Brca1^−/−^* MEFs [25], [26], loss of p53 fully rescued the growth defect of *Rnf8^−/−^* MEFs. Interestingly, primary *Rnf8^−/−^* MEFs display similar characteristics to those observed with *Brca1^−/−^* MEFs, mainly a senescent phenotype, increased basal levels of p21 and p53 but no increase of p19^ARF^ [48]. These results suggest that rescue of the senescent phenotype in *Rnf8^−/−^p53^−/−^* MEFs is caused by an abrogation of the p53-p21 pathway.

Our studies of *Rnf8^−/−^p53^−/−^* mice characterized p53-dependent and independent roles for defective homeostasis of *Rnf8^−/−^* immune cells and the post-natal growth defects of *Rnf8^−/−^* mice [14]. In contrast to *53bp1^−/−^* thymocytes [49], but similar to *Brca1* null thymocytes [25], loss of p53 fully rescued thymocyte numbers in *Rnf8^−/−^* mice. These data are consistent with the higher steady state level of p53 in *Rnf8^−/−^* thymocytes compared to *WT* controls [14], and suggest that impaired homeostasis of thymocytes in *Rnf8^−/−^* mice is p53-dependent. In addition, as in Brca1 deficient splenocytes [25], loss of p53 rescued the lymphopenia associated with Rnf8 deficiency [14], [16]. Notably, loss of p53 failed to rescue the growth defects of *Rnf8^−/−^* mice and the reduced bone marrow cellularity, suggesting that they occur in p53-independent manner. Previous studies have shown that defects in B and T-cell progenitors did not necessarily affect cellularity of the thymus and the spleen. In *Foxo3^−/−^* knockout mice the number of pre B-cells and recirculating B-cells were diminished in the bone marrow but the number of B-cells in the spleen remained the same [50]. In addition, it was shown that although T-cell progenitors were unable to settle in the thymus of *Ccr7^−/−^Ccr9^−/−^* double knockout mice, thymocyte numbers of these mutants remained close to those found in *WT* mice [51]. Therefore, it is possible that while B-cell progenitors are reduced in the bone marrow of *Rnf8^−/−^p53^−/−^* mice, there is increased expansion and a rescue of the B-cell population in spleen of these mice due to absence of p53-dependent apoptosis.

While CSR has been reported to be defective in the presence of impaired DSB signaling as in the case of the absence of Rnf8 [14], [16], Rnf168 [28], [52], H2ax [30] or 53bp1 [31], [32]; loss of p53 does not affect B-cell class switching to IgG1 [33]. Our data indicate that *Rnf8^−/−^p53^−/−^* B-cells display similarly reduced CSR level to what was observed with *Rnf8^−/−^* B-cells. These data suggest that the impaired CSR associated with Rnf8 loss is p53-independent. The inability of p53 to rescue CSR defects suggests that even if *Rnf8^−/−^p53^−/−^* B-cells escape the apoptosis associated with Rnf8 deficiency, they are still unable to undergo CSR. This might be because Rnf8 plays a direct role in the repair of the DSBs at switch junctions as was previously suggested [14], [16]. This is similar to what has been observed for other mutations of DNA repair proteins involved in CSR such as DNA-PKcs and Artemis [53], [54].

The repair of DSBs is mediated by the HR or the NHEJ repair pathways [55]. Previous studies indicated that loss of Rnf8 impairs both repair pathways [12], [56]. Rnf8 has also been reported to mediate ubiquitylation and proteasomal degradation of the NHEJ protein Ku80 and this function has been proposed to be important for the role Rnf8 plays in NHEJ [12]. While defects of HR or NHEJ increase cancer risks, additional loss of p53 further exacerbates these risks [55], [57]. Consistent with its role in DSB signaling and repair, loss of Rnf8 leads to increased genomic instability and cancer risks [14]. *Rnf8^−/−^p53^−/−^* B-cells display a considerable increase in genomic instability compared to either *Rnf8^−/−^* or *p53^−/−^* B-cells under both untreated and irradiated conditions. This could be explained by the fact that cells with elevated levels of genomic instability, caused by unrepaired DSBs, are not eliminated in the absence of p53 and are allowed to accumulate genomic lesions without undergoing programmed cell death. The very high levels of genomic instability in *Rnf8^−/−^p53^−/−^* mice led us to hypothesize that they will develop tumors with shorter latency compared to single mutant mice. Indeed, *Rnf8^−/−^p53^−/−^* mice developed thymic and B-cell lymphomas and very rapidly succumb to their tumors.

Interestingly, even though Rnf8 and Rnf168 have been proposed to function in the same DNA damage signaling pathway, the tumor spectrum of *Rnf8^−/−^p53^−/−^* mice was different from what was observed in *Rnf168^−/−^p53^−/−^* mice which develop primarily B-cell lymphomas, followed by thymomas and sarcomas [28]. In addition, consistent with the increased cancer risk in Rnf8 but not Rnf168 deficient background [14], [28], *Rnf8^−/−^p53^−/−^* mice developed tumors with a significantly shorter latency compared to *Rnf168^−/−^p53^−/−^* mice. The impaired checkpoints in *Rnf8^−/−^p53^−/−^* cells and their elevated levels of spontaneous and IR-induced genomic instability compared to *p53^−/−^* and *Rnf8^−/−^* cells are likely to play important role in the increased propensity of these cells to undergo tumorigenic transformation.

The tumor spectrum observed with *Rnf8^−/−^p53^+/−^* mice, which included thymomas, lymphomas, rhabdomyosarcoma, adenocarcinoma and osteoma, was different from what we observed for *Rnf8^−/−^p53^−/−^* mice. In addition, while *Rnf8^−/−^p53^+/−^* mice started to die at 90 days of age with 80% dead by 485 days, consistent with previous studies [43], *p53^+/−^* mice started to die at around 9 months of age and only 50% of them were dead by 18 months of age. Thus, loss of Rnf8 in a p53 heterozygous background also leads to decreased latency period and increased penetrance of tumors. LOH of p53 is likely to play an important role in tumorigenesis in *Rnf8^−/−^p53^+/−^* mice as the majority of tumors from these mice showed loss of the *WT* p53 allele.

Collectively, our data indicate that Rnf8 and p53 work synergistically to activate checkpoints, maintain genomic integrity and suppress cancer development. While p53-independent mechanisms contribute to some of the defects associated with Rnf8 deficiency, the accumulation of DNA double strand breaks in the absence of Rnf8, and the subsequent activation of p53, likely play a major role in the mechanisms that eliminate damaged cells through cell cycle arrest, blunted growth or increased cell death. Thus, when p53 is deficient, uncontrolled growth and proliferation of damaged *Rnf8^−/−^* cells and their dramatic increase of genomic instability drive the remarkably high tumor incidence in these mice.

## Materials and Methods

### Mice

*Rnf8^−/−^* mice generated using the AS0574 ES clone [14] and *p53^−/−^* mice [58] (Taconic), both in a mixed 129/J×C57BL/6 genetic background, were crossed to generate *Rnf8^+/−^p53^+/−^* mice. These mice were then bred to obtain *Rnf8^−/−^p53^−/−^* mutants. Mice were genotyped by PCR using the following primers: forward *WT Rnf8* 5′-TGATGACACCTGGGCATGT-3′; reverse *WT Rnf8* 5′-TCTTTGAGACAGCGCCTGG-3′; forward mutant *Rnf8* 5′-TCAAAGGTTTGCCCTCTCTGAT-3′; reverse mutant *Rnf8* 5′-CGGAGCGGATCTCAAACTCT-3′; forward *WT p53* 5′-GTGTTTCATTAGTTCCCCACCTTGAC-3′; reverse *WT p53* 5′-ATGGGAGGCTGCCAGTCCTAACCC-3′; forward *p53* mutant 5′-TTTACGGAGCCCTGGCGCTCGATGT-3′; reverse *p53* mutant 5′-GTGGGAGGGACAAAAGTTCGAGGCC -3′. All animal experiments were done in compliance with the Ontario Cancer Institute animal care committee guidelines. Animal protocols were approved by the Animal Resource Center of Ontario Cancer Institute.

### Western blot analysis

Equal amounts of cell lysates were loaded onto 7.5% or 10% polyacrylamide gels and were transferred onto PVDF membrane. Detection of protein was done using primary antibodies against phosphorylated p53-Ser15 (Cell Signaling), p53 (FL393, Santa Cruz Biotechnology), p21 (M-19, Santa Cruz Biotechnology), p19 (Novus), β-actin (Santa Cruz Biotechnology) and HRP-conjugated secondary antibodies against rabbit or rat.

### Senescence-associated β-galactosidase assay

Passage 3 primary MEFs were fixed and stained using the Senescence β-galactosidase staining kit from Cell Signaling according to Manufacturer's instructions. The cells were visualized using a brightfield Olympus CKX41 microscope using the 10× objective and images were taken using the Infinity 2 camera and Infinity Capture software.

### Immune cell counts and flow cytometry

Thymocytes, splenocytes and bone marrow cells were isolated from 6 weeks old mice. Red blood cells collected with splenocytes and bone marrow cells were lysed using red blood cell lysis buffer (Sigma). Cells were counted and stained with anti-B220, anti-TCRβ, anti-CD4, anti-CD8, anti-IgM, anti-IgD and anti-CD43 antibodies (eBioscience) and were examined using a FACSCalibur flow cytometer (Becton Dickinson). The data was analyzed using the FlowJo software (Tree Star, Inc.).

### Cell death assays

Freshly isolated thymocytes and splenocytes from 6 weeks old mice were irradiated with 2 Gy ionizing radiation and allowed to recover for 12 and 24 hours respectively. Cells were stained with PI (Sigma) and flow cytometry analysis was performed using a FACSCalibur flow cytometer. The data was analyzed using the FlowJo software.

### *In vitro* proliferation assays

*WT*, *Rnf8^−/−^*, *p53^−/−^* and *Rnf8^−/−^p53^−/−^* MEFs were generated from E13.5 embryos. Passage 2 MEFs were used to generate growth curves by 3T3 passaging. Briefly, 3×10^5^ cells were seeded in a 60 mm dish and allowed to grow for 3 days. The cells were trypsinized and the same number of cells was reseeded on the plate. This was repeated until cells became senescent.

### Class switch recombination

Negative selection, using the mouse B-cell negative isolation kit (Invitrogen), was used to purify B-cells from freshly isolated splenocytes of 6–8 week old mice. Cells were stained with CFSE (Invitrogen) and allowed to proliferate for 4 days in RPMI medium in the presence of anti-CD40 (eBioscience) and IL-4 (eBioscience). Cells were then stained with anti-B220 (eBioscience) and IgG1 (eBioscience) antibodies and analyzed by flow cytometry.

### G1-S checkpoint assay

Passage 1 or 2 primary MEFs were either untreated or irradiated (10 Gy) and left to recover for 16 hours before pulsing with 10 µM BrDU for 4 hours. Cells were then fixed overnight with 70% ethanol before staining with FITC-conjugated anti-BrDU antibody (e-Bioscience) and PI as previously described [59].

### G2-M checkpoint assay

Passage 1–3 MEFs were irradiated with 2 Gy ionizing radiation (IR) or left untreated. 1 hour after the irradiation cells were fixed with 70% ethanol overnight and were then stained with FITC- conjugated anti-phospho histone H3 (Ser10) antibody (Cell Signaling) and PI and analysed by flow cytometry as previously described [59].

### Immunofluorescence

Early passage primary MEFs were plated on glass coverslips. The cells were then left untreated or irradiated with 8 Gy IR. Cells were fixed in 4% paraformaldehyde for 20 minutes and incubated overnight with anti-γ-H2ax (07-164, Millipore; 1∶500 dilution), anti-Mdc1 (A300-757A, Bethyl, 1∶500 dilution), anti-Brca1 (1∶500 dilution) and anti-53bp1 (A300-272A, Bethyl; 1∶500 dilution) in dilution buffer (5% FBS, 3% BSA, 0.05% Triton X-100 in PBS). Following washes with 10% dilution buffer and PBS, cells were incubated with a goat anti-rabbit Alexa fluor 488-conjugated antibody (A11008, Molecular Probes; 1∶1000 dilution). After washing, cells were stained with DAPI (Invitrogen) and mounted on slides using Mowiol (Sigma). Cells were visualized and quantified for their foci using a Leica DM 4000 B microscope with a 100× oil immersion objective. Image acquisition and overlay was performed using the Leica Application Suite V 4.0 software (Leica Microsystems, Switzerland).

### Chromosomal aberration analysis

Freshly isolated splenocytes from 6-week-old tumor-free mice were stimulated to proliferate for 24 hours in RPMI medium containing 10 µg/ml LPS (Sigma). Cells were then either left untreated or irradiated with 2 Gy IR. 24 hours post-irradiation, cells were incubated in hypotonic buffer (75 mM potassium chloride solution) at 37°C and then fixed with ice-cold 3∶1 methanol-acetic acid solution. Chromosome numbers and aberrations were scored for at least 50 metaphases from two separate sets of samples.

### Tumor analysis

Cohorts of *Rnf8^−/−^p53^−/−^*, *Rnf8^−/−^p53^+/−^*, *Rnf8^−/−^*, *p53^−/−^*, *p53^+/−^* and *WT* mice were monitored for a period of one year. Moribund mice were sacrificed and examined for the presence of tumors. Tumors of lymphoid origin were characterized using flow cytometry. Histopathological examination of H&E stained sections of tumors embedded in paraffin was performed using a Leica DM 4000 B microscope and images were acquired using the Leica Application Suite software. Kaplan-Meier plots for tumor free survival of mice were charted. Log-Rank test statistical analysis was performed using Prism 5 (GraphPad Software, Inc.).

### Immunohistochemistry

Tissues fixed in buffered formalin and embedded in paraffin and sectioned at 5 µm were stained using anti-p53 antibody (FL393, Santa Cruz Biotechnology) or anti- cleaved caspase-3 antibody (Cell Signaling). Slides were viewed using a Leica DM 4000 B microscope and images were acquired using the Leica Application Suite software.

### Southern blot analysis of p53 loss of heterozygosity

Genomic DNA isolated from tails and tumors of *Rnf8^−/−^p53^+/−^* mice were digested overnight with the restriction enzyme BamH1 and run on a 0.8% agarose gel. DNA were then transferred to a Hybond N^+^ membrane (GE healthcare) and hybridized with a ^32^P-labeled *p53* specific probe. The bands were visualized using a phosphoimager (Typhoon 9410, Amersham Biosciences).

### Statistical analysis

The data is presented as the mean ± SD. Statistical analysis of experimental data was performed using student t-test and statistical significance was determined at P<0.05. The log-rank test was used to generate tumor-free survival curves for mouse cohorts. Two-sided Fisher exact test using the Mstat software was used to determine statistical significance for analysis of chromosomal aberrations.

## Supporting Information

Figure S1 Loss of p53 does not rescue the growth defects of *Rnf8^−/−^* mice. (A) Body weight was measured for *Rnf8^−/−^p53^−/−^* (n = 7), *Rnf8^−/−^* (n = 3), *p53^−/−^* (n = 4) and *WT* (n = 5) 6-week-old female littermates. (B) Body weight was measured for *Rnf8^−/−^p53^−/−^* (n = 5), *Rnf8^−/−^* (n = 9), *p53^−/−^* (n = 4) and *WT* (n = 5) 6-week-old male littermates. * denotes P<0.05 using a student t-test.
(TIF)

Figure S2 Characterization of thymocyte and bone marrow subpopulations of *Rnf8^−/−^p53^−/−^* mice. (A) Thymocytes from 6 week-old *Rnf8^−/−^p53^−/−^* and control littermates were stained with anti-CD8 and anti-TCRβ and the proportion of each subpopulation was determined by flow cytometry. (B) Bone marrow cells from 6 week-old *Rnf8^−/−^p53^−/−^* mice and control littermates were stained with anti-B220 and anti-IgM and bone marrow subpopulations were determined by flow cytometry.
(TIF)

Figure S3 Increased p53 expression and apoptosis levels in *Rnf8^−/−^* thymus and spleen. (A) p53 IHC staining of spleen sections of *Rnf8^−/−^* mice and *WT* littermates. (B) Quantification of p53 positive cells in spleen. An average of 20 randomly chosen fields was counted at 63× magnifications. (C) Anti-cleaved caspase-3 IHC staining of spleen of *Rnf8^−/−^p53^−/−^*, *Rnf8^−/−^*, *p53^−/−^* and *WT* mice. (D) Quantification of cleaved caspase-3 positive cells in the spleen. An average of 20 fields were counted at 63× magnification. Data is representative of 3 different experiments. * indicates statistical significance (P<0.05). Bar: 50 µm.
(TIF)

Figure S4 Tissue-specific increase of p53 levels in *Rnf8^−/−^* mice. (A) p53 IHC staining of various organs from *Rnf8^−/−^* mice and their control littermates. Only basal cells of intestinal crypts show stronger p53 staining in *Rnf8^−/−^* mice compared to *WT* littermates. (B) Quantification is shown for p53-positive cells in intestinal crypts. These data are representative of three different experiments. * indicates p<0.05. Bar: 100 µm.
(TIF)

Figure S5 *Rnf8^−/−^p53^−/−^* MEFs display impaired recruitment of 53bp1 to DNA double strand break sites. *Rnf8^−/−^p53^−/−^* primary MEFs and their controls were left untreated (UT) or irradiated with 8 Gy and allowed to recover for 0.5, 4 and 24 hours before fixation. Cells were stained using anti-53bp1 antibody and counterstained with DAPI. Representative images of cells stained with anti-53bp1from at least three independent experiments. h: hour.
(TIF)

Figure S6 *Rnf8^−/−^p53^−/−^* MEFs display increased basal and residual Mdc1 foci. Mdc1 staining for *Rnf8^−/−^p53^−/−^* and control MEFs. *Rnf8^−/−^p53^−/−^* primary MEFs and their controls were left untreated (UT) or irradiated with 8 Gy and allowed to recover for 0.5, 6 and 24 h before fixation. Cells were stained using anti-Mdc1 antibody and counterstained with DAPI. Representative images of cells stained with anti-Mdc1 from at least three independent experiments. h: hour. Quantification of cells with >10 foci is shown. Three repeats of this experiment have been done. * denotes statistical significance (P<0.05).
(TIF)

Figure S7 *Rnf8^−/−^p53^−/−^* MEFs display impaired Brca1 recruitment to break sites. Brca1 staining of *Rnf8^−/−^p53^−/−^* and control MEFs. *Rnf8^−/−^p53^−/−^* early 3T3 MEFs and their controls were left untreated (UT) or irradiated with 8 Gy and allowed to recover for 6 and 24 hours before fixation. Cells were stained using anti-Brca1 antibody and counterstained with DAPI. Quantification of cells with >10 foci in *Rnf8^−/−^p53^−/−^* and control MEFs 6 and 24 hrs post-irradiation is shown. Three repeats of this experiment have been done. * denotes statistical significance (P<0.05).
(TIF)

Figure S8 Representative flow cytometry analysis of *Rnf8^−/−^p53^−/−^* tumors. (A) Flow cytometry analysis of *Rnf8^−/−^p53^−/−^* B-cell lymphomas stained with anti-B220, anti-Thy1.2 and anti-IgM antibodies. (B, C) Flow cytometry analysis of *Rnf8^−/−^p53^−/−^* thymomas stained with anti-TCRβ, anti-CD4 and anti-CD8 antibodies.
(TIF)

Table S1 Cumulative cell number in *Rnf8^−/−^p53^−/−^* and control MEFs after 15 days in culture. Cumulative cell numbers for *Rnf8^−/−^p53^−/−^* and control MEFs during 3T3 passaging. The average cumulative cell number ± SD of at least 4 different experiments is shown for each time point.
(DOC)
