# Supplementary figures and images for: Synergistic Interaction of Rnf8 and p53 in the Protection against Genomic Instability and Tumorigenesis
Source: PLoS Genet. 2013 Jan 31;9(1):e1003259. doi: 10.1371/journal.pgen.1003259 (PMC3561120; doi:10.1371/journal.pgen.1003259)

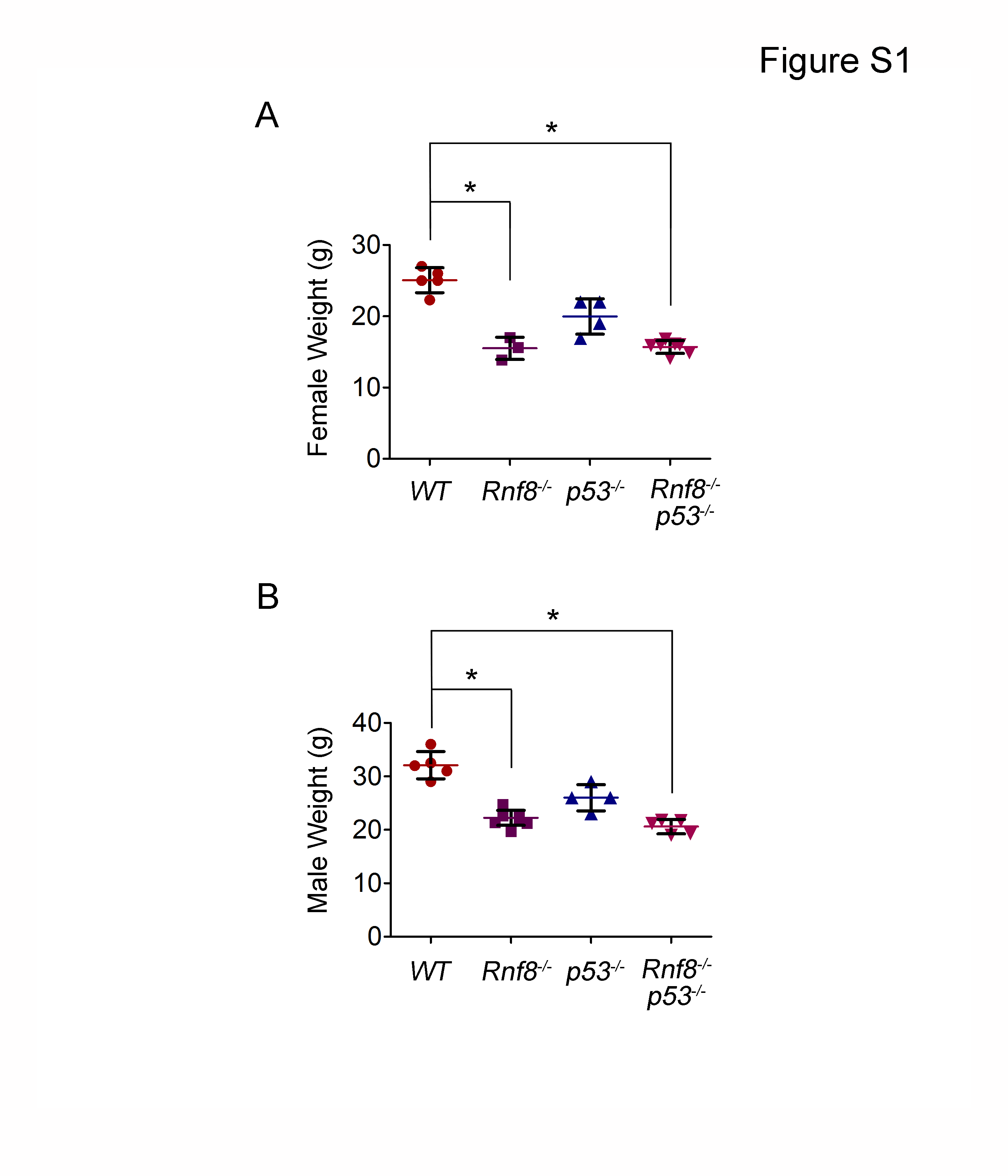

Supplement: Figure S1 — Loss of p53 does not rescue the growth defects of Rnf8−/− mice. (A) Body weight was measured for Rnf8−/−p53−/− (n = 7), Rnf8−/− (n = 3), p53−/− (n = 4) and WT (n = 5) 6-week-old female littermates. (B) Body weight was measured for Rnf8−/−p53−/− (n = 5), Rnf8−/− (n = 9), p53−/− (n = 4) and WT (n = 5) 6-week-old male littermates. * denotes P<0.05 using a student t-test. (TIF) [file pgen.1003259.s001.tif]

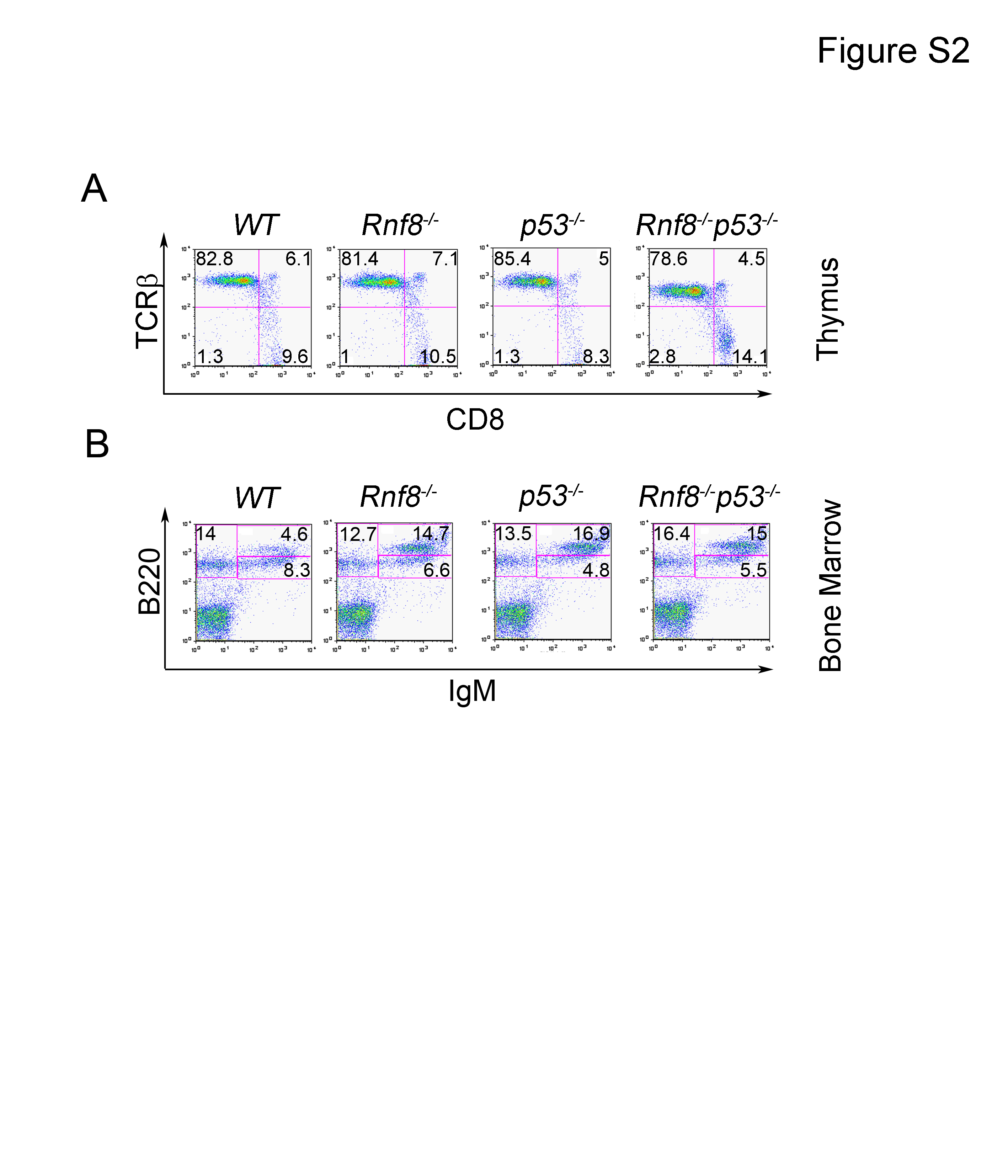

Supplement: Figure S2 — Characterization of thymocyte and bone marrow subpopulations of Rnf8−/−p53−/− mice. (A) Thymocytes from 6 week-old Rnf8−/−p53−/− and control littermates were stained with anti-CD8 and anti-TCRβ and the proportion of each subpopulation was determined by flow cytometry. (B) Bone marrow cells from 6 week-old Rnf8−/−p53−/− mice and control littermates were stained with anti-B220 and anti-IgM and bone marrow subpopulations were determined by flow cytometry. (TIF) [file pgen.1003259.s002.tif]

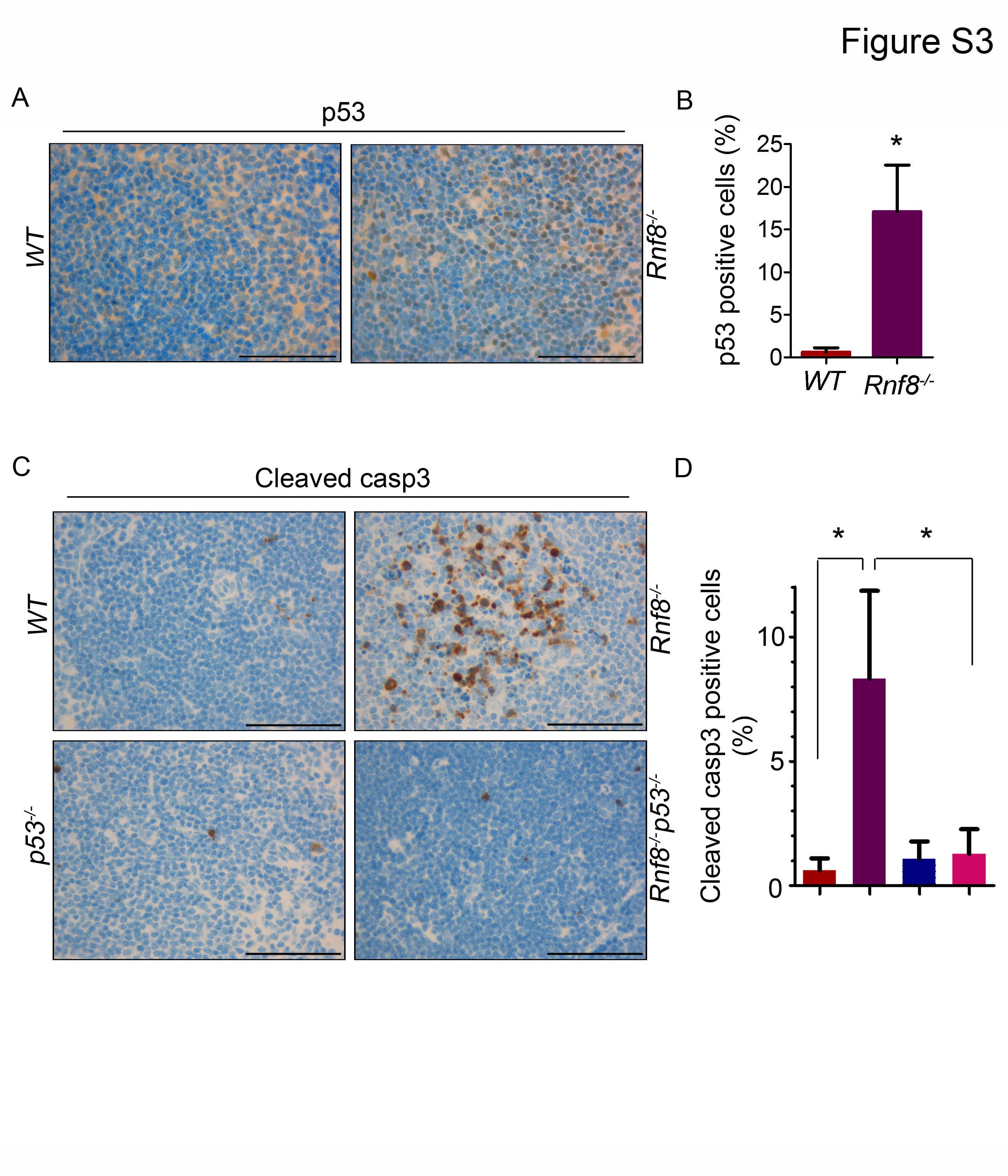

Supplement: Figure S3 — Increased p53 expression and apoptosis levels in Rnf8−/− thymus and spleen. (A) p53 IHC staining of spleen sections of Rnf8−/− mice and WT littermates. (B) Quantification of p53 positive cells in spleen. An average of 20 randomly chosen fields was counted at 63× magnifications. (C) Anti-cleaved caspase-3 IHC staining of spleen of Rnf8−/−p53−/−, Rnf8−/−, p53−/− and WT mice. (D) Quantification of cleaved caspase-3 positive cells in the spleen. An average of 20 fields were counted at 63× magnification. Data is representative of 3 different experiments. * indicates statistical significance (P<0.05). Bar: 50 µm. (TIF) [file pgen.1003259.s003.tif]

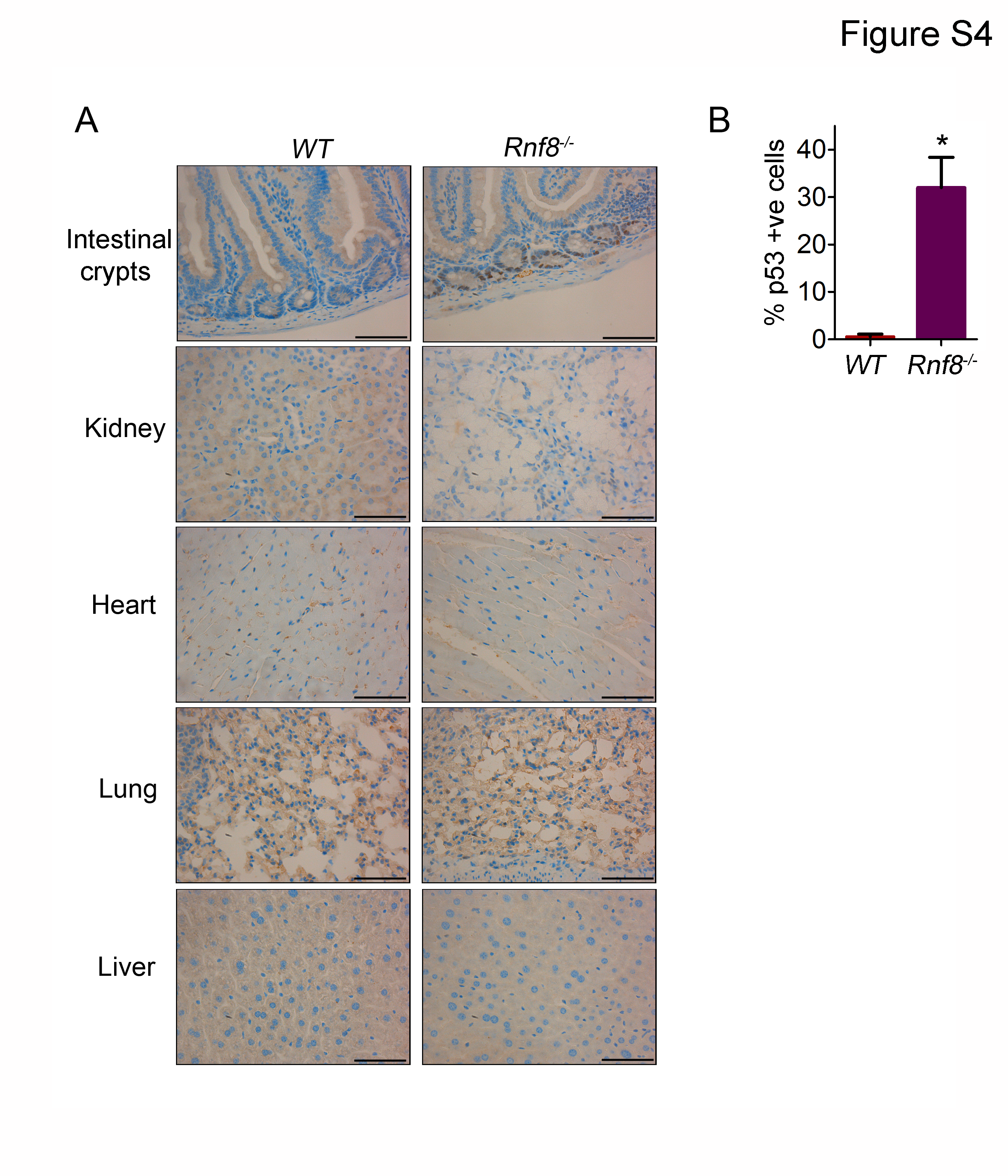

Supplement: Figure S4 — Tissue-specific increase of p53 levels in Rnf8−/− mice. (A) p53 IHC staining of various organs from Rnf8−/− mice and their control littermates. Only basal cells of intestinal crypts show stronger p53 staining in Rnf8−/− mice compared to WT littermates. (B) Quantification is shown for p53-positive cells in intestinal crypts. These data are representative of three different experiments. * indicates p<0.05. Bar: 100 µm. (TIF) [file pgen.1003259.s004.tif]

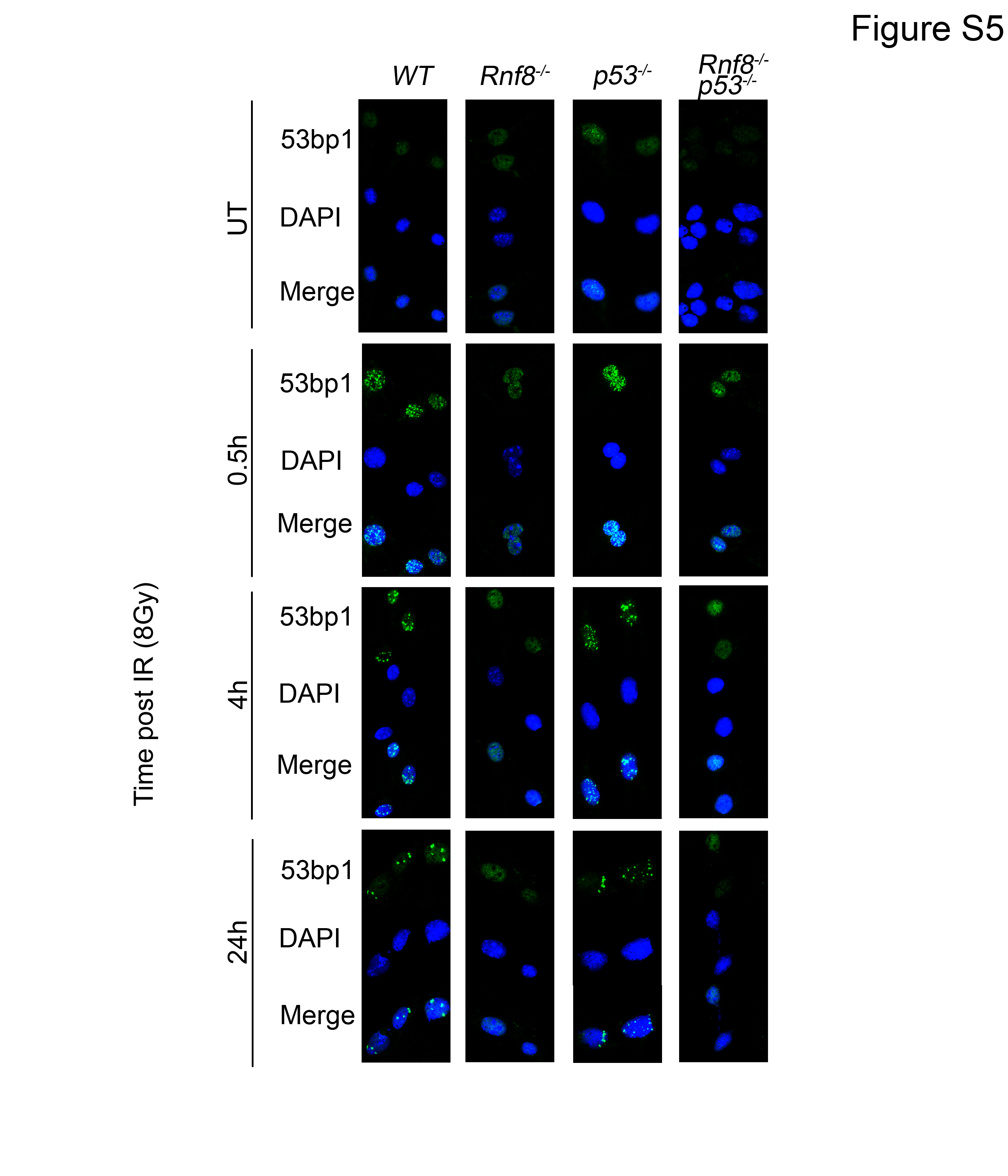

Supplement: Figure S5 — Rnf8−/−p53−/− MEFs display impaired recruitment of 53bp1 to DNA double strand break sites. Rnf8−/−p53−/− primary MEFs and their controls were left untreated (UT) or irradiated with 8 Gy and allowed to recover for 0.5, 4 and 24 hours before fixation. Cells were stained using anti-53bp1 antibody and counterstained with DAPI. Representative images of cells stained with anti-53bp1from at least three independent experiments. h: hour. (TIF) [file pgen.1003259.s005.tif]

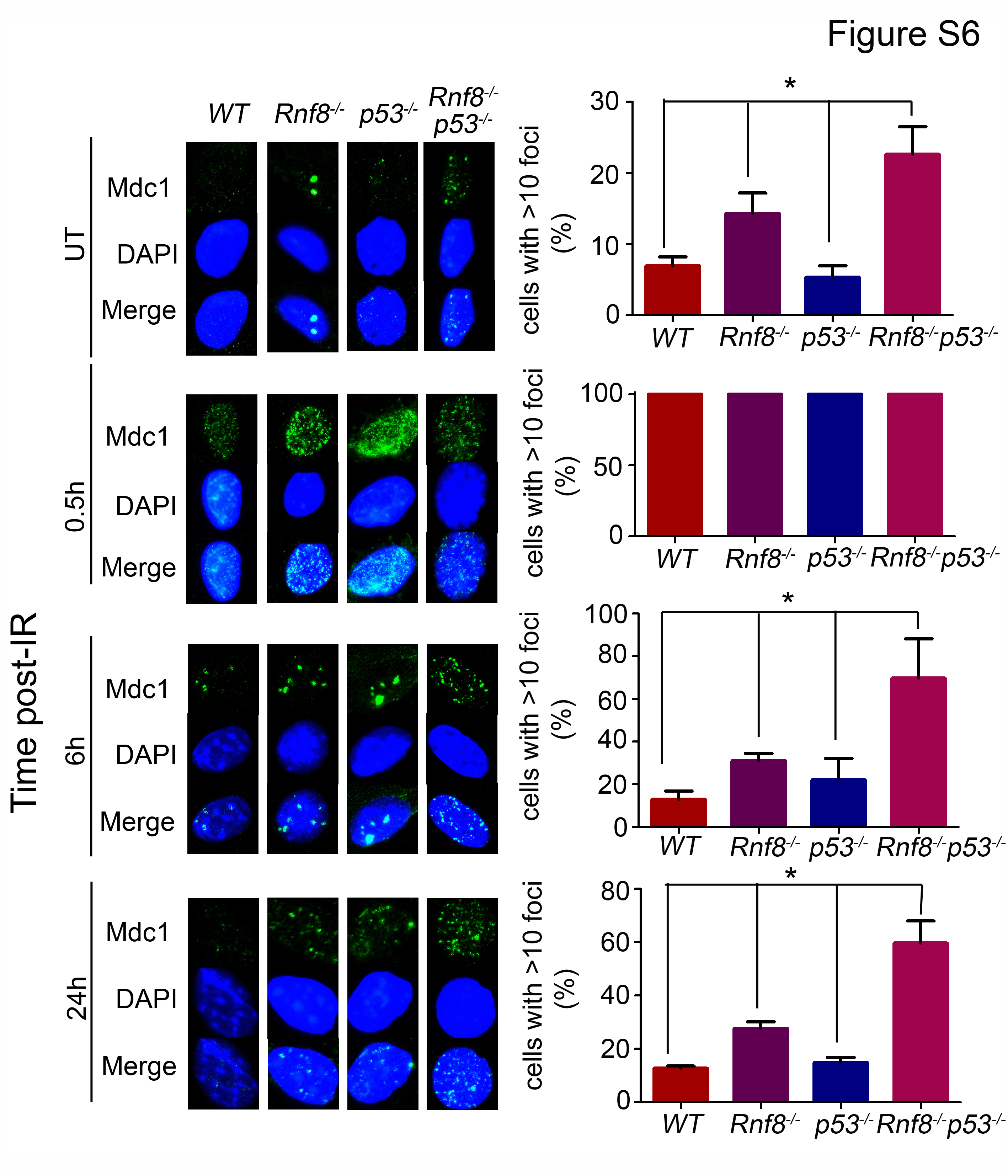

Supplement: Figure S6 — Rnf8−/−p53−/− MEFs display increased basal and residual Mdc1 foci. Mdc1 staining for Rnf8−/−p53−/− and control MEFs. Rnf8−/−p53−/− primary MEFs and their controls were left untreated (UT) or irradiated with 8 Gy and allowed to recover for 0.5, 6 and 24 h before fixation. Cells were stained using anti-Mdc1 antibody and counterstained with DAPI. Representative images of cells stained with anti-Mdc1 from at least three independent experiments. h: hour. Quantification of cells with >10 foci is shown. Three repeats of this experiment have been done. * denotes statistical significance (P<0.05). (TIF) [file pgen.1003259.s006.tif]

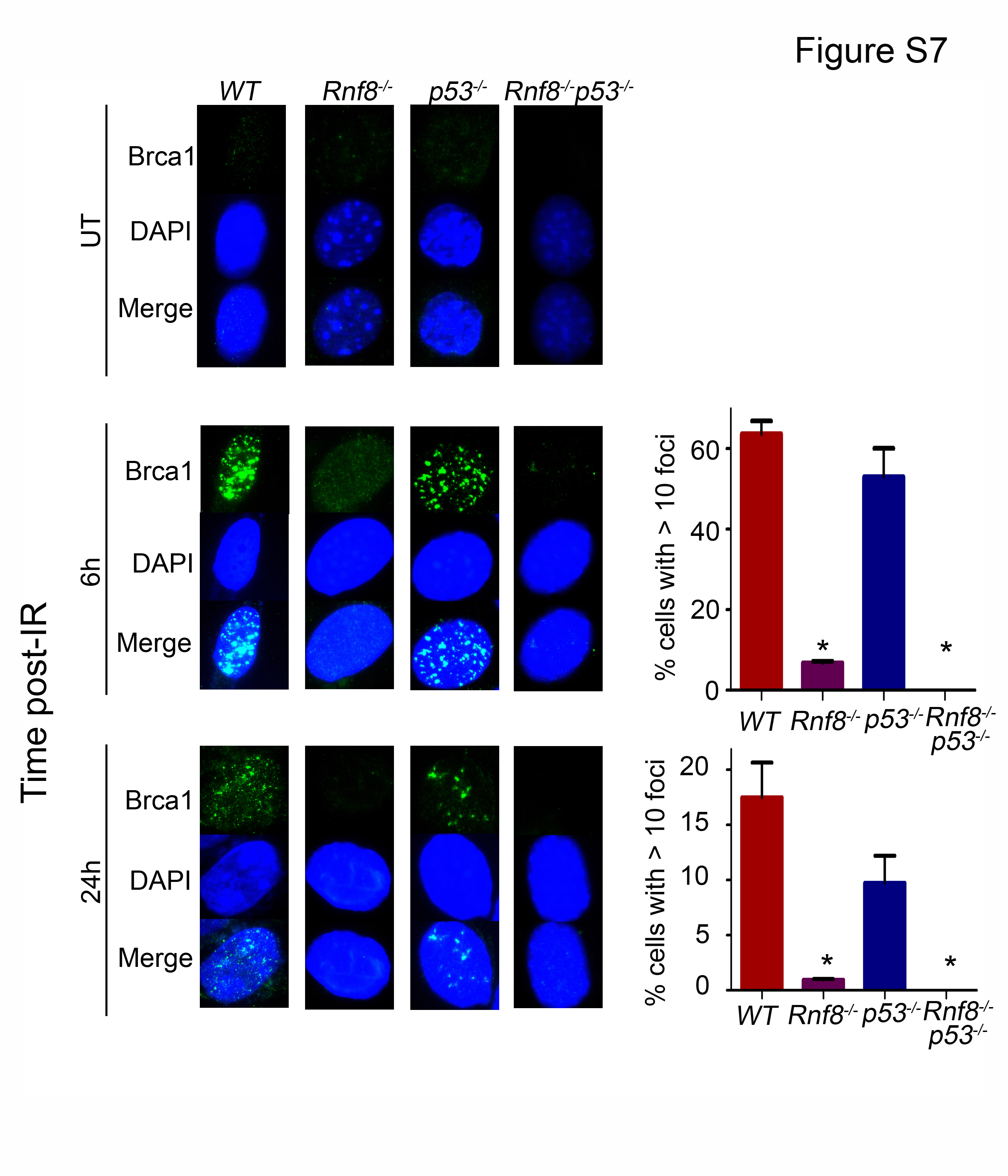

Supplement: Figure S7 — Rnf8−/−p53−/− MEFs display impaired Brca1 recruitment to break sites. Brca1 staining of Rnf8−/−p53−/− and control MEFs. Rnf8−/−p53−/− early 3T3 MEFs and their controls were left untreated (UT) or irradiated with 8 Gy and allowed to recover for 6 and 24 hours before fixation. Cells were stained using anti-Brca1 antibody and counterstained with DAPI. Quantification of cells with >10 foci in Rnf8−/−p53−/− and control MEFs 6 and 24 hrs post-irradiation is shown. Three repeats of this experiment have been done. * denotes statistical significance (P<0.05). (TIF) [file pgen.1003259.s007.tif]

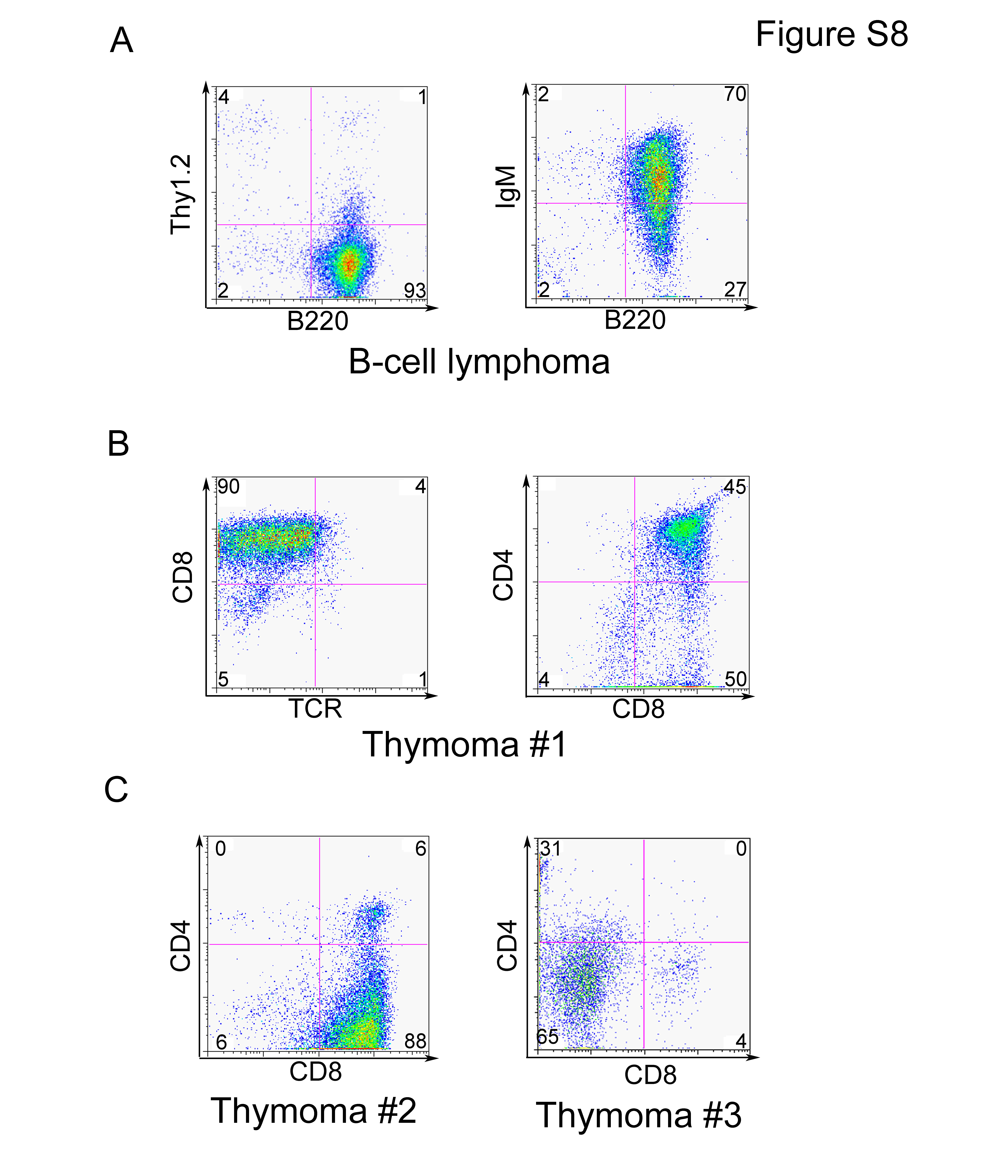

Supplement: Figure S8 — Representative flow cytometry analysis of Rnf8−/−p53−/− tumors. (A) Flow cytometry analysis of Rnf8−/−p53−/− B-cell lymphomas stained with anti-B220, anti-Thy1.2 and anti-IgM antibodies. (B, C) Flow cytometry analysis of Rnf8−/−p53−/− thymomas stained with anti-TCRβ, anti-CD4 and anti-CD8 antibodies. (TIF) [file pgen.1003259.s008.tif]
