# Supplementary material for: Synergistic Interaction of Rnf8 and p53 in the Protection against Genomic Instability and Tumorigenesis
Source: PLoS Genet. 2013 Jan 31;9(1):e1003259. doi: 10.1371/journal.pgen.1003259 (PMC3561120; doi:10.1371/journal.pgen.1003259)
Supplement: Table S1 — Cumulative cell number in Rnf8−/−p53−/− and control MEFs after 15 days in culture. Cumulative cell numbers for Rnf8−/−p53−/− and control MEFs during 3T3 passaging. The average cumulative cell number ± SD of at least 4 different experiments is shown for each time point. (DOC) [file pgen.1003259.s009.doc]

| Days | *WT* | *Rnf8^-/-^* | *p53^-/-^* | *Rnf8^-/-^p53^-/-^* |
| --- | --- | --- | --- | --- |
| 0 | 0.3 x10^6^ | 0.3 x10^6^ | 0.3 x10^6^ | 0.3 x10^6^ |
| 3 | 0.71 ± 0.2 x10^6^ | 0.53 ± 0.2x10^6^ | 0.9 ± 0.1 x10^6^ | 1.29 ± 0.3 x10^6^ |
| 6 | 1.24 ± 0.5 x10^6^ | 0.9 ± 0.4 x10^6^ | 2.8 ± 0.9 x10^6^ | 6.2 ± 2 x10^6^ |
| 9 | 2.5 ± 0.9 x10^6^ | 1.3 ± 0.5 x10^6^ | 9.6 ± 4.1 x10^6^ | 23.4 ± 12 x10^6^ |
| 12 | 4.1 ± 1.2 x10^6^ | 1.3 ± 0.8 x10^6^ | 31 ± 17 x10^6^ | 94 ± 48 x10^6^ |
| 15 | 5.2 ± 1.4 x10^6^ | 1.2 ± 0.5 x10^6^ | 69 ± 33 x10^6^ | 370 ± 240 x10^6^ |

**Table S1**: Cumulative cell number in *Rnf8^-/-^p53^-/-^* and control MEFs after 15 days in cell culture
